# Sugar-modified G-quadruplexes: effects of LNA-, 2′F-RNA– and 2′F-ANA-guanosine chemistries on G-quadruplex structure and stability

**DOI:** 10.1093/nar/gkt1312

**Published:** 2013-12-25

**Authors:** Zhe Li, Christopher Jacques Lech, Anh Tuân Phan

**Affiliations:** School of Physical and Mathematical Sciences, Nanyang Technological University, Singapore 637371

## Abstract

G-quadruplex-forming oligonucleotides containing modified nucleotide chemistries have demonstrated promising pharmaceutical potential. In this work, we systematically investigate the effects of sugar-modified guanosines on the structure and stability of a (4+0) parallel and a (3+1) hybrid G-quadruplex using over 60 modified sequences containing a single-position substitution of 2′-O-4′-C-methylene-guanosine (^LNA^G), 2′-deoxy-2′-fluoro-riboguanosine (^F^G) or 2′-deoxy-2′-fluoro-arabinoguanosine (^FANA^G). Our results are summarized in two parts: (I) Generally, ^LNA^G substitutions into ‘anti’ position guanines within a guanine-tetrad lead to a more stable G-quadruplex, while substitutions into ‘syn’ positions disrupt the native G-quadruplex conformation. However, some interesting exceptions to this trend are observed. We discover that a ^LNA^G modification upstream of a short propeller loop hinders G-quadruplex formation. (II) A single substitution of either ^F^G or ^FANA^G into a ‘syn’ position is powerful enough to perturb the (3+1) G-quadruplex. Substitution of either ^F^G or ^FANA^G into any ‘anti’ position is well tolerated in the two G-quadruplex scaffolds. ^FANA^G substitutions to ‘anti’ positions are better tolerated than their ^F^G counterparts. In both scaffolds, ^FANA^G substitutions to the central tetrad layer are observed to be the most stabilizing. The observations reported herein on the effects of ^LNA^G, ^F^G and ^FANA^G modifications on G-quadruplex structure and stability will enable the future design of pharmaceutically relevant oligonucleotides.

## INTRODUCTION

G-quadruplexes are four-stranded nucleic acid structures composed of stacked layers of guanine tetrads, stabilized by Hoogsteen hydrogen bonds and coordinating cations (1,2). Guanine-rich G-quadruplex-forming sequences are present in some critical regions of the human genome, and the formation of these structures has been shown to play important roles in various biological processes (3–10).

From a therapeutic perspective, many engineered G-quadruplex–forming sequences show high affinity towards biologically important protein targets. For example, G-quadruplex–forming oligonucleotides have been discovered with anti-coagulant, anti-cancer and anti-HIV activity (11–16). However, native DNA chemistry is prone to enzymatic digestion. The incorporation of alternative nucleic acid chemistries can enhance the lifetime and other pharmacological properties of G-quadruplex-based drugs.

Modification of the base (17–20) or phosphate–sugar backbone (20–26) can have beneficial effects on the stability, kinetics, resistance to enzymatic digestion and cellular uptake of biologically active G-quadruplexes. For example, past studies have investigated the effects of introducing modified base and sugar-backbone chemistries into the thrombin-binding aptamer (TBA) (19), known for its anti-coagulant properties. The use of modified chemistries in the TBA has lead to higher stability (22,25,27–31), increased binding affinity (25,30) and enhanced biological activity including studies *in vivo* (28,30,32–34). In a similar manner, modified nucleic acid chemistries have been used to enhance the pharmacological properties of anti-HIV aptamers (25,35–40).

**Figure 1.**
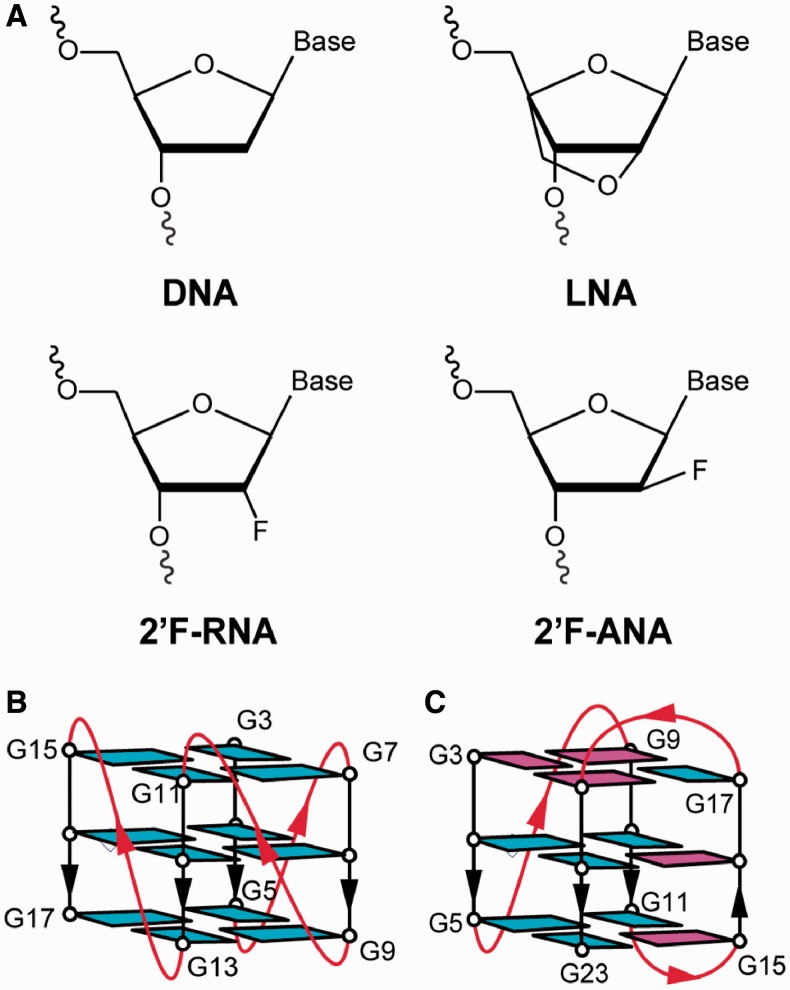
Modified nucleotides and G-quadruplex scaffolds used in this study: (**A**) Sugar chemistries of DNA, LNA, 2′F-RNA and 2′F-ANA. Schematic structures of the (**B**) (4+0) parallel G-quadruplex formed by d[T_2_(G_3_T)_4_] and the (**C**) (3+1) hybrid G-quadruplex formed by d[T_2_(G_3_T_2_A)_3_G_3_A]. Guanines are coloured based on their ‘syn’ (magenta) or ‘anti’ (cyan) conformation.

One alternative DNA chemistry that has received notable attention is Locked Nucleic Acid (LNA) (41), a ribonucleotide analogue with a 2′-O-4′-C-methylene linkage (Figure 1A). Introduction of LNA can improve oligonucleotide stability towards enzymatic digestion as well as the thermal stability of duplexes and triplexes (41,42). Previous studies have shown that LNA modifications can greatly enhance the RNA cleaving rate of a DNAzyme (43). Additionally, LNA is generally soluble in water and non-toxic (44,45). In the context of G-quadruplexes, it has been reported that the introduction of LNA-modified guanosine (^LNA^G) stabilizes the tetrameric G-quadruplexes formed by the d[T^LNA^G_3_T], d[T(G^LNA^G)_2_T] and d[T^LNA^G_4_T] sequences (46,47). ^LNA^G has been previously observed to favour an ‘anti’ glycosidic conformation of the base (48), and studies have taken advantage of this preference to engineer the G-quadruplex folding topology (49,50). Substitutions of ^LNA^G into positions that adopt a ‘syn’ conformation tend to push structural equilibrium towards a parallel G-quadruplex where all guanines adopt an ‘anti’ conformation (48,49,51). Incorporation of ^LNA^G has also been used to enhance the inhibitory properties of biologically active G-quadruplex molecules (28,40).

The sugar-modified nucleotides 2′-deoxy-2′-fluoro-riboguanosine (^F^G) and 2′-deoxy-2′-fluoro-arabinoguanosine (^FANA^G) represent another useful family of chemical tools, containing a proton to fluorine modification at the C2′ position of the sugar (Figure 1A). These chemistries have shown promise for increasing the stability and anti-sense potency of duplexes formed by short interference RNAs (52,53). 2′F-RNA and 2′F-ANA modified nucleotides have been observed to increase the resistance of modified oligonucleotides to degradation by nuclease (25,54,55). In the context of G-quadruplex, modification with 2′F-ANA nucleotides has allowed for enhanced G-quadruplex aptamer stability and nuclease resistance (25). Recent works from our laboratory and others have also shown that the ^F^G and ^FANA^G chemistries can be used to manipulate the folding topology of G-quadruplexes (26,56).

Considering the demonstrated potential of sugar-modified nucleotides for enhancing the drug-like properties of G-quadruplexes, we set out on a systematic study to characterize the effects of ^LNA^G, ^F^G and ^FANA^G incorporation into two G-quadruplex DNA scaffolds: (i) The first scaffold is an intramolecular ‘(4+0)’ parallel-stranded (PS) G-quadruplex formed by the ‘PS-series’ sequence d[T_2_(G_3_T)_4_] (57). This structure contains all strands oriented in the same direction connected by single-nucleotide (1-nt) propeller loops, with all guanine bases adopting an ‘anti’ glycosidic conformation (Figure 1B). (ii) The second scaffold is an intramolecular ‘(3+1)’ hybrid G-quadruplex formed by the human telomeric (HT) sequence d[T_2_(G_3_T_2_A)_3_G_3_A] termed the ‘HT-series’ (58). This structure contains three strands oriented in one direction and the fourth in the opposite direction (Figure 1C). The strands are connected by edgewise and propeller loops that are three nucleotides (3-nt) in length. Furthermore, guanine bases within the G-tetrad core adopt a mixture of ‘syn’ and ‘anti’ glycosidic conformations. These two G-quadruplex scaffolds were chosen for study as they have been well characterized and represent distinct types of folding topologies, which exhibit a variety of different structural features.

In order to investigate how to effectively incorporate ^LNA^G, ^FANA^G and ^F^G nucleotides into G-quadruplex structures, we characterize the conformation and thermal stability of G-quadruplexes formed by modified sequences using a combination of nuclear magnetic resonance (NMR), ultraviolet (UV) absorption and circular dichroism (CD) spectroscopic methods. Our systematic study reveals important considerations for use of these sugar-modified chemical tools within G-quadruplex nucleic acids. Such knowledge will enable the rational design of engineered G-quadruplexes containing these chemistries for pharmaceutical and nanotechnology applications.

## MATERIALS AND METHODS

### Sample preparation

Oligonucleotides were chemically synthesized using an Applied Biosystems 394 DNA/RNA synthesizer (Foster City, CA, USA). ^LNA^G phosphoramidite was purchased from ScienceWerke (Singapore). Sugar-modified ^F^G and ^FANA^G phosphoramidites were purchased from Glen Research (Sterling, VA, USA). Oligonucleotides were purified using a Poly-Pak cartridge (Glen Research) following standard protocol. After purification, samples were dialysed successively against water, KCl solution (25 mM) and water again.

After lyophilization, DNA was dissolved and stored in potassium phosphate (KPi) buffer (pH 7) containing 10% D_2_O and 20 μM 4,4-dimethyl-4-silapentane-1-sulfonic acid (DSS). The concentrations of potassium in DNA stocks were as follows: 5 mM KPi for ^LNA^G-modified PS-series, 20 mM KPi for ^LNA^G-modified HT-series, 1 mM KPi for ^F^G-modified and ^FANA^G-modified PS-series, and 5 mM KPi supplemented with 5 mM KCl for ^F^G-modified and ^FANA^G-modified HT-series.

### Nuclear magnetic resonance

NMR experiments were performed on a 600 or 700 MHz Bruker NMR spectrometer (Billerica, MA, USA) at 25°C using jump-and-return-type pulse sequences for water suppression (61,62). Chemical shifts were calibrated by DSS in NMR buffer. Samples were annealed by heating at 100°C for 5 min and cooled down slowly overnight before recording NMR spectra and subsequent CD studies. Samples for 1D NMR experiments were examined in DNA stock conditions described above with DNA strand concentrations as follows: ^LNA^G PS-series 0.15 mM; ^LNA^G HT-series 0.1–0.2 mM; ^F^G and ^FANA^G PS-series 0.05 mM; ^F^G and ^FANA^G HT-series 0.05–0.4 mM.

### Circular Dichroism

CD experiments were performed on a JASCO-815 spectropolarimeter (Tokyo, Japan) using a 1-cm path length quartz cuvette. CD spectra were taken at 20°C in a range of 220–320 nm and accumulated over three scans for the ^LNA^G PS-series and 10 scans for all other sequences. Spectra were baseline corrected and zero-corrected at 320 nm. CD spectra were normalized by DNA strand concentration determined by concurrent UV absorbance measurements.

Samples for the ^LNA^G PS-series were prepared by diluting the DNA stock with 1 mM KPi to obtain DNA concentration of 3 µM and KPi concentration of 1.1 mM. Samples for the ^LNA^G HT-series contained a DNA concentration of 10 µM in the stock salt conditions stated above. Samples for ^F^G and ^FANA^G modified PS-series and HT-series contained DNA strand concentration of 4–6 μM with stock salt conditions described in above section. DNA concentration was expressed in strand molarity with the extinction coefficient of modified sequences approximated to that of the unmodified sequence.

### Thermal stability measurements

CD melting was performed for the ^LNA^G-modified PS-series and loop-elongated samples by monitoring CD intensity at 260 nm against temperature over a range of 44–93°C. CD melting was performed by heating the samples at 93°C for 20 min and then cooling to 44°C at a rate of 0.2°C/min. Samples were then heated up again to 93°C at the same rate. For several samples, this range was extended to a lower temperature to monitor complete melting transitions. The melting temperature (T_m_) values were obtained by baseline normalization of CD melting curves to create folded fraction curves (59,60). The T_m_ value is determined to be the temperature at which half of the sample is in the folded state. The T_m_ values presented are an average over cooling and heating curves.

UV melting experiments were performed for all other samples on a JASCO V-650 UV-Vis spectrophotometer (Tokyo, Japan). UV melting experiments were conducted by monitoring the UV absorbance at 295 nm (59) using a 1-cm path length quartz cuvette over a temperature range of 15–84°C for the HT-series and 43–89°C for the PS-series. This range was extended for several sequences to monitor the complete transition. UV melting was done by first heating the samples to their maximum temperature for 30 min, then cooling down to the minimum temperature at a rate of 0.1°C/min. Samples were then heated up to the maximum temperature at the same rate. The UV absorbance of samples was recorded at 295 nm and corrected by subtracting the absorbance at 320 nm as well as baseline corrected with a reference cell. Fraction folded curves were calculated in the manner described above. Samples were prepared as described in ‘Circular Dichroism’ section.

The effects of sugar-modifed guanosine substitutions were studied in low-salt conditions to monitor the full denaturation of these samples in thermal stability experiments.

## RESULTS AND DISCUSSION

G-quadruplex formation of sugar-modified sequences (Table 1 and 2) was evaluated by monitoring the imino proton region (10.5–12.5 ppm) of ^1^H NMR spectra (63). Peaks in this region can be used to determine the number of G-quadruplex conformations present. High similarity between NMR spectra is a strong indicator that sequences adopt the same folding topology. Validating the folding topology of modified sequences is important, given that small changes in sequence can have large effects on the conformations adopted by G-quadruplex–forming sequences (64,65). CD spectroscopy was also used to probe the G-quadruplex folding topology based on well-characterized patterns in CD spectra (66). The thermal stability of sugar-modified sequences was evaluated through a series of UV and CD melting experiments. The T_m_ values of sequences are presented as a useful quantitative measure for comparing the thermal stability of modified G-quadruplexes. We also present thermodynamic parameters ΔH, ΔS and ΔG for modified sequences in Supplementary Data.

**Table 1.** LNA-modified sequences used in this study and their thermal stability

Name^a^	Sequence (5′→3′)^b^	*T*_m_(°C)^c^	Δ*T*_m_(°C)
(4+0) native	TTGGGTGGGTGGGTGGGT	77.1 ± 0.5	–
PS-L3	TT**L**GGTGGGTGGGTGGGT	82.0 ± 0.4	4.9
PS-L4	TTG**L**GTGGGTGGGTGGGT	85.3 ± 0.3	8.1
PS-L5	TTGG**L**TGGGTGGGTGGGT	36.0 ± 0.4	−41.2
PS-L7	TTGGGT**L**GGTGGGTGGGT	84.3 ± 0.0	7.2
PS-L8	TTGGGTG**L**GTGGGTGGGT	80.2 ± 0.2	3.1
PS-L9	TTGGGTGG**L**TGGGTGGGT	32.2 ± 0.1	−45.0
PS-L11	TTGGGTGGGT**L**GGTGGGT	84.9 ± 0.1	7.8
PS-L12	TTGGGTGGGTG**L**GTGGGT	79.8 ± 0.1	2.7
PS-L13	TTGGGTGGGTGG**L**TGGGT	31.7 ± 0.5	−45.5
PS-L15	TTGGGTGGGTGGGT**L**GGT	83.2 ± 0.1	6.1
PS-L16	TTGGGTGGGTGGGTG**L**GT	81.0 ± 0.1	3.8
PS-L17	TTGGGTGGGTGGGTGG**L**T	80.4 ± 0.1	3.3
(3+1) native	TTGGGTTAGGGTTAGGGTTAGGGA	57.4 ± 0.2	–
HT-L3	TT**L**GGTTAGGGTTAGGGTTAGGGA	55.4 ± 0.3	−2.0
HT-L4	TTG**L**GTTAGGGTTAGGGTTAGGGA	61.6 ± 0.0	4.2
HT-L5	TTGG**L**TTAGGGTTAGGGTTAGGGA	56.8 ± 0.4	−0.6
HT-L9	TTGGGTTA**L**GGTTAGGGTTAGGGA	–	–
HT-L10	TTGGGTTAG**L**GTTAGGGTTAGGGA	59.3 ± 0.5	1.9
HT-L11	TTGGGTTAGG**L**TTAGGGTTAGGGA	58.3 ± 0.3	0.9
HT-L15	TTGGGTTAGGGTTA**L**GGTTAGGGA	–	–
HT-L16	TTGGGTTAGGGTTAG**L**GTTAGGGA	–	–
HT-L17	TTGGGTTAGGGTTAGG**L**TTAGGGA	55.1 ± 0.4	−2.3
HT-L21	TTGGGTTAGGGTTAGGGTTA**L**GGA	–	–
HT-L22	TTGGGTTAGGGTTAGGGTTAG**L**GA	–	–
HT-L23^d^	TTGGGTTAGGGTTAGGGTTAGG**L**A	60.5 ± 0.1	3.1

^a^The ‘HT-series’ denotes sequences modified from the (3+1) G-quadruplex-forming sequence, while the ‘PS-series’ denotes sequences modified from the (4+0) G-quadruplex-forming sequence.

^b^Residues with LNA-modified guanosine are denoted as (**L**).

^c^Thermal stability data were obtained via UV melting (HT-series) and CD melting (PS-series) experiments. Salt conditions were 20 mM KPi for the HT-series and 1.1 mM KPi for the PS-series. Thermal stability data for the HT-series is presented for sequences that demonstrate a single major conformation in NMR spectra. The uncertainties (±values) indicate the hysteresis between heating and cooling curves. Information regarding thermodynamic parameters ΔH, ΔS and ΔG are presented in Supplementary Table S1.

^d^Sequence contains a small secondary melting transition at the low temperature range.

### Part I: LNA-guanosine

#### Substitution of ^LNA^G into a (4+0) parallel G-quadruplex: LNA modifications are detrimental when substituted before short propeller loops

**Figure 2.**
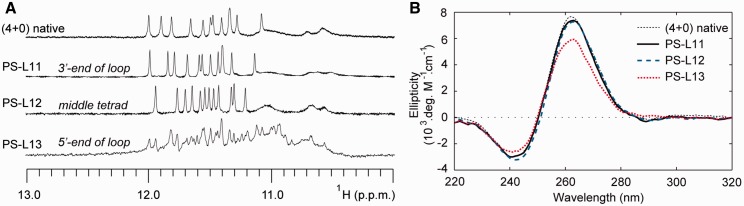
(**A**) NMR imino proton spectra and (**B**) CD spectra of select PS-series sequences containing single-position ^LNA^G substitutions on the 3′-end of a loop, middle tetrad or 5′-end of a loop.

**Figure 3.**
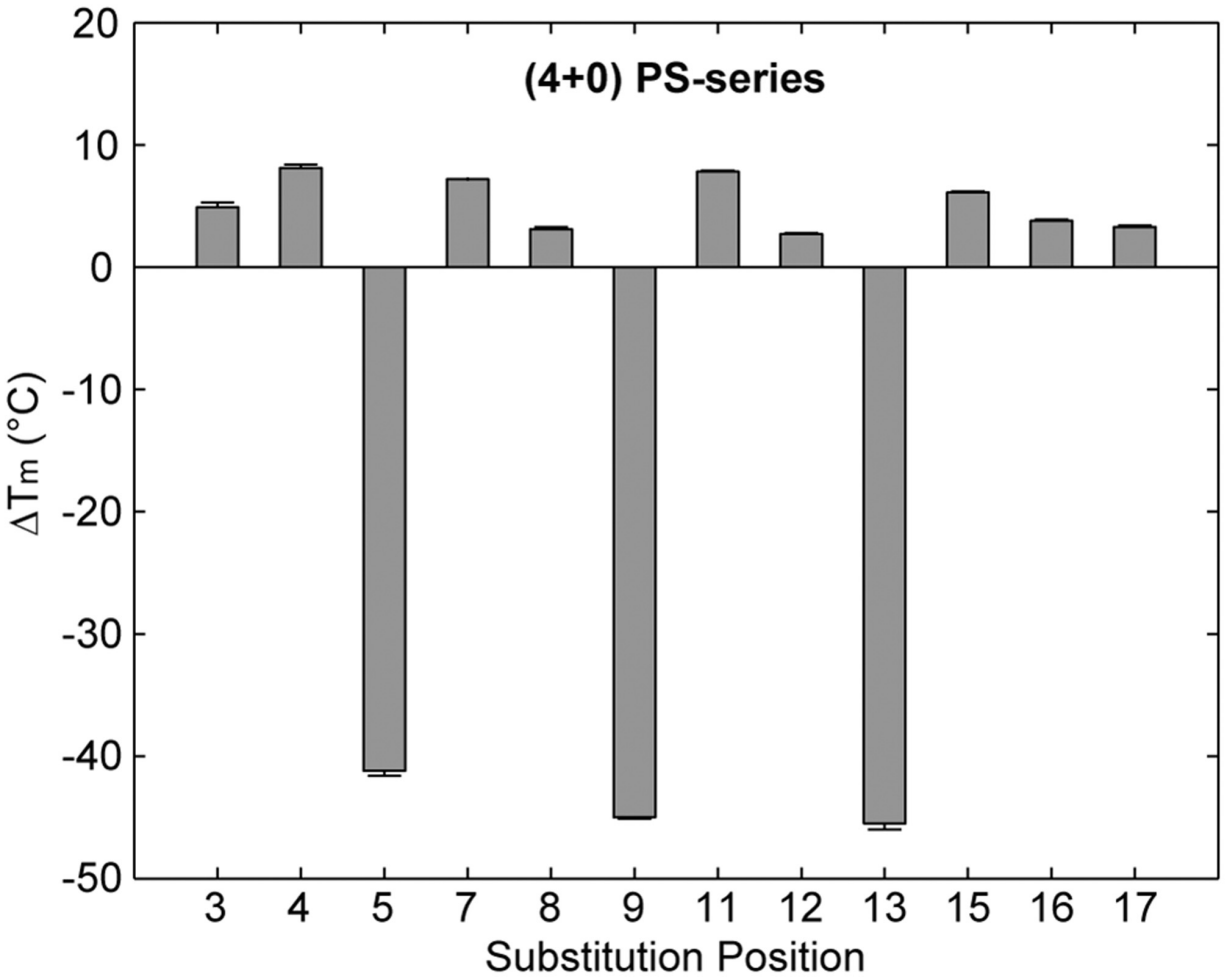
ΔT_m_ of the PS-series sequences containing single-position ^LNA^G modifications with respect to that of the native sequence, as determined by CD melting experiments.

Within the PS-series, 9 of the 12 single-position ^LNA^G-modified sequences were observed by NMR to form a single major G-quadruplex conformation and gave imino proton spectra which were similar to the native sequence (Figure 2A and Supplementary Figure S1). CD spectra of these sequences were characteristic of a (4+0) parallel G-quadruplex conformation, with a maximum at ∼260 nm and a minimum at ∼240 nm (Figure 2B and Supplementary Figure S3). Conversely, substitution of ^LNA^G into positions G5, G9 and G13 lead to multiple conformations in NMR spectra and a reduction in the intensity of CD spectra at 260 nm (Figure 2). Sequences that formed multiple conformations also demonstrated a large decrease in thermal stability with a drop in melting temperature of >40°C (Figure 3 and Supplementary Figures S5–S6). Alternatively, sequences that formed a single conformation displayed an increase in T_m_ with changes in melting temperature (ΔT_m_) between +2.7°C and +8.1°C.

**Figure 4.**
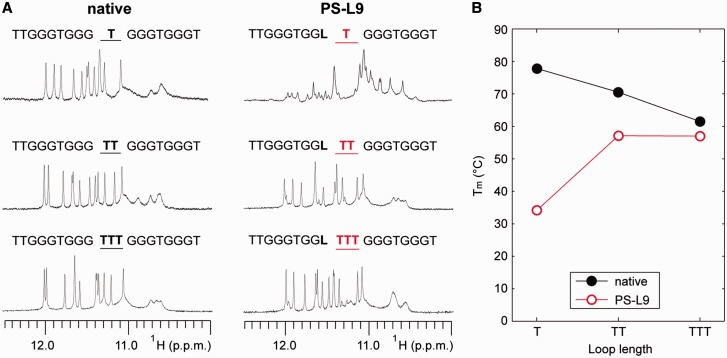
Effect of loop length on the compatibility of LNA incorporation preceding a propeller loop. (**A**) NMR imino proton spectra of (4+0) G-quadruplex–forming sequences with the central loop of different length. The length of the central 1-nt T10 loop of the native DNA sequence and the modified PS-L9 sequence containing a LNA substitution at position 9 were varied, resulting in sequences containing a loop T, TT or TTT. (**B**) The T_m_ value of the native and PS-L9 sequences with different loop lengths.

We note that the unfavourable substitutions at positions G5, G9 and G13 are located upstream and adjacent to 1-nt propeller loops within this intramolecular (4+0) G-quadruplex scaffold (Figure 1B). Our results suggest the incompatibility of LNA incorporation into residues preceding short propeller loops. Considering this observation, we proceeded to explore the role of the loop length in the destabilization induced by a LNA modification upstream of propeller loops. We expanded the central 1-nt loop at position T10 to two (TT) and three (TTT) nucleotides for both the native and the ^LNA^G9-modified PS-L9 sequence. The effects of loop elongation were monitored using NMR and CD melting experiments (Figure 4). While NMR spectra indicated a single conformation for all three native sequences, the emergence of a single major conformation was only observed for LNA-modified sequences with the central loop longer than 1-nt. The T_m_ values of the loop-extended native and LNA-modified PS-L9 sequences converged as the loop length increased. The expansion of the T10 loop in the PS-L9 sequence by a single thymine led to a dramatic recovery of the thermal stability, with an increase in T_m_ of 23°C. The extension of this loop by a third thymine led to a relatively small increase in stability. At a loop length of 3-nt, the difference in T_m_ between the loop-extended native and LNA-modified PS-L9 sequences converged to a mere 4.5°C compared with a 43.6°C difference in sequences containing a 1-nt loop.

In this study, we show that ^LNA^G substitution upstream of short propeller loops strongly destabilizes G-quadruplex structure. ^LNA^G substitution to positions G5, G9 and G13 in the (4+0) G-quadruplex is observed to be disruptive, despite the ‘anti’ glycosidic conformation of the substituted guanine base. Elongation of these propeller loops is shown to reduce the detrimental effects of ^LNA^G substitutions. It is likely that the conformational constraints on the sugar-backbone geometry imposed by the short propeller loop are incompatible with the conformationally restricted LNA sugar-backbone. The loop-dependent nature of ^LNA^G substitutions into ‘anti’-position guanines is in direct contrast to the ^F^G and ^FANA^G nucleotides discussed in Part II. The disruptive effect of ^LNA^G-modification at the 5′-end of short loops constitutes an important consideration for future design of G-quadruplexes containing LNA modifications.

#### Substitution of ^LNA^G into a (3+1) hybrid G-quadruplex: LNA modifications generally affect structure in a syn/anti dependent manner

**Figure 5.**
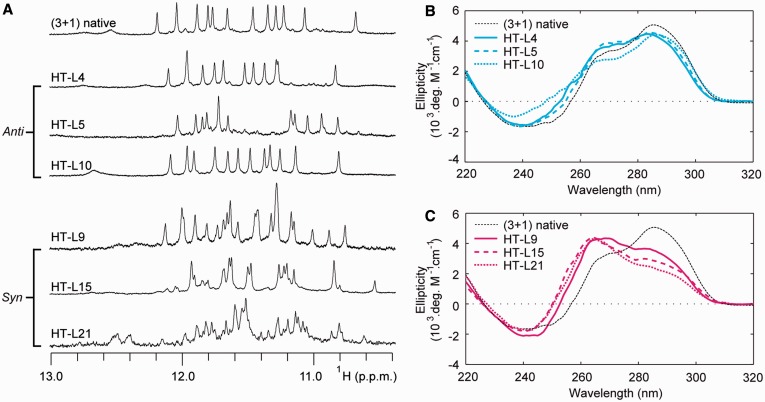
The ‘syn’/‘anti’ preference of LNA substitutions in the HT-series. (**A**) NMR imino proton spectra and (**B**–**C**) CD spectra are shown for select sequences containing single-position LNA substitutions to (B) ‘anti’ positions and (C) ‘syn’ positions.

Similar to our study of the (4+0) G-quadruplex scaffold, we systematically substituted ^LNA^G into guanines within the (3+1) G-quadruplex structure formed by the HT-series, which contains guanine residues in both ‘syn’ and ‘anti’ conformations (Figure 1C). Within the HT-series, 7 of the 12 modified sequences demonstrated a single major conformation as monitored by NMR spectra (Figure 5A and Supplementary Figure S2). CD spectra of these sequences displayed the profile of a (3+1) G-quadruplex, similar to that of the native sequence (Figure 5B and Supplementary Figure S4). Conversely, the other five ^LNA^G substitutions induced multiple conformations as observed in NMR spectra (Figure 5A). The CD spectra of these five modified sequences exhibit a profile that deviates from that of the native (3+1) G-quadruplex, with an increase in intensity at 260 nm and a decrease at 295 nm (Figure 5C).

**Figure 6.**
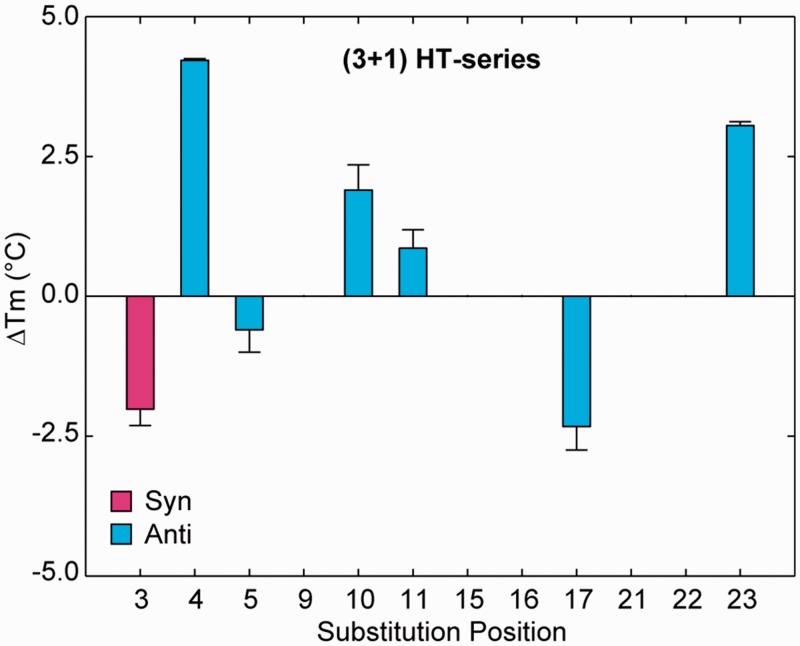
ΔT_m_ of HT-series sequences containing single-position ^LNA^G modifications. Values are plotted for sequences containing single-position ^LNA^G substitutions that demonstrated a single conformation. Error bars indicate the hysteresis between heating and cooling melting curves.

Among the seven modified sequences displaying a single (3+1) G-quadruplex conformation, six sequences involved a ^LNA^G substitution to a guanine in an ‘anti’ glycosidic conformation, and surprisingly, one involved a ^LNA^G substitution to ‘syn’ guanine G3. Substitution with ^LNA^G produced mixed effects on the thermal stability of modified sequences (Figure 6 and Supplementary Figures S7 and S8). Substitution into ‘anti’ guanines led to ΔT_m_ values in the range of −2.3°C to +4.2°C. Substitution to the ‘syn’-position G3 led to a destabilization, with a ΔT_m_ of −2.0°C. Among the five ^LNA^G substitutions that induced multiple conformations, four were made to ‘syn’-position guanines and one was made to ‘anti’-position G22. Interpretation of T_m_ values for sequences that adopt multiple species is complicated by complex melting curves and uncertainty about which structural transition is being analysed. Therefore we do not discuss the T_m_ values of these sequences in detail.

It is generally observed that substitution of ^LNA^G into ‘syn’-position guanines within the HT-series disrupted the folding topology of the G-quadruplex and led to the coexistence of multiple species, while ‘anti’ positions substitutions are generally tolerated. This is in agreement with the findings of previous studies (46–48,50,51). Despite the general ‘syn’/‘anti’ dependence of the ^LNA^G modifications, we note some important exceptions to this trend. As discussed in the above section, substitutions into ‘anti’ guanines directly upstream of 1-nt propeller loops were highly disruptive within the PS-series (Figures 2 and 3). Interestingly, ^LNA^G modification to G5, G11 and G17 within the HT-series, located upstream of a 3-nt propeller or edgewise loop, were the least favourable ‘anti’-position substitutions tested in the (3+1) G-quadruplex with ΔT_m_ values of −0.6°C, +0.9°C and −2.3°C, respectively (Figure 6). Additionally, a substitution of ^LNA^G into an ‘anti’-conformation guanine before a 2-nt edgewise loop has been previously reported to destabilize the TBA (28). These data suggest that ^LNA^G substitutions into ‘anti’-position guanines before many types of loops of short to medium length may have an adverse effect on G-quadruplex stability.

**Figure 7.**
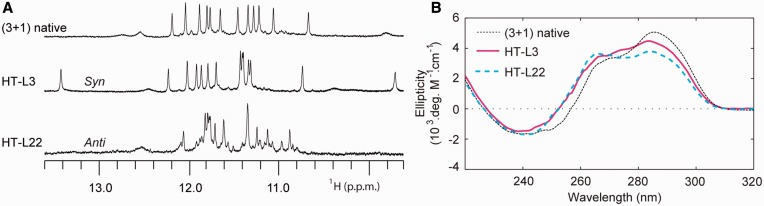
(**A**) NMR imino proton spectra and (**B**) CD spectra are presented for the ‘syn’-position modified HT-L3 sequence and the ‘anti’-position modified HT-L22 sequence.

Other exceptions to the ‘syn’/‘anti’ dependence of ^LNA^G modifications are also observed. Within the (3+1) G-quadruplex, ^LNA^G substitution to ‘anti’-position G22 is observed to disrupt the native conformation (Figure 7). It is not yet clear why G22 does not tolerate ^LNA^G modification in this (3+1) G-quadruplex conformation. Additionally, a single instance of ‘syn’-position substitution to residue G3 unexpectedly resulted in a presumably undisrupted (3+1) G-quadruplex conformation (Figure 7). The NMR spectrum of this sequence contains sharp peaks at 9.7 and 13.4 ppm not present in the native (3+1) G-quadruplex, suggesting enhanced external base pairing (Figure 7A). However, this ‘syn’-substitution comes at a cost of reduced thermal stability (Figure 6). The exceptions we observe to the generalized ‘syn’/‘anti’ dependence of ^LNA^G substitutions illustrates the need for cautious substitution of this sugar-modified chemistry into G-quadruplex nucleic acids.

### Part II: 2′F-RNA- and 2′F-ANA-guanosine

#### 2′F-RNA and 2′F-ANA modifications destabilize G-quadruplex DNA in syn positions and are universally tolerated in anti positions

**Figure 8.**
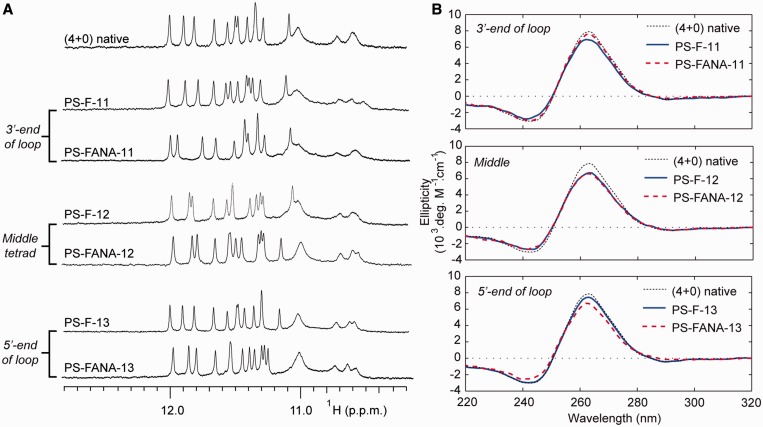
Incorporation of ^F^G and ^FANA^G substitutions into the (4+0) G-quadruplex scaffold: (**A**) NMR and (**B**) CD spectra of select PS-series samples containing single ^F^G and ^FANA^G substitutions to guanines located in a variety of structural environments. ^F^G and ^FANA^G are well tolerated in all positions of the PS-series.

Within the (4+0) G-quadruplex, substitutions were made into eight ‘anti'- position guanines, G3-G5, G8, G11-G13 and G17, to explore a variety of structural environments including guanines adjacent to loops, guanines within the central tetrad layer and guanines toward the flanking ends of the sequence. All of the ^F^G and ^FANA^G modifications into the (4+0) G-quadruplex demonstrated highly similar NMR and CD spectra compared with the unmodified sequence with no sign of disruption to G-quadruplex conformation (Figure 8).

**Table 2.** 2′F-RNA- and 2′F-ANA-modified sequences used in this study and their thermal stability

Name^a^	Sequence (5′→3′)^b^	T_m_(°C)^c^	ΔT_m_(°C)
(4+0) native	TTGGGTGGGTGGGTGGGT	76.5 ± 0.4	
PS-F-3	TT**F**GGTGGGTGGGTGGGT	76.8 ± 0.1	0.3
PS-F-4	TTG**F**GTGGGTGGGTGGGT	77.0 ± 0.0	0.5
PS-F-5	TTGG**F**TGGGTGGGTGGGT	75.4 ± 0.1	−1.1
PS-F-8	TTGGGTG**F**GTGGGTGGGT	76.9 ± 0.1	0.3
PS-F-11	TTGGGTGGGT**F**GGTGGGT	77.2 ± 0.2	0.7
PS-F-12	TTGGGTGGGTG**F**GTGGGT	75.4 ± 0.1	−1.1
PS-F-13	TTGGGTGGGTGG**F**TGGGT	76.6 ± 0.3	0.1
PS-F-17	TTGGGTGGGTGGGTGG**F**T	75.2 ± 0.0	−1.4
PS-FANA-3	TT**F**GGTGGGTGGGTGGGT	77.1 ± 0.2	0.6
PS-FANA-4	TTG**F**GTGGGTGGGTGGGT	79.7 ± 0.0	3.2
PS-FANA-5	TTGG**F**TGGGTGGGTGGGT	77.2 ± 0.3	0.6
PS-FANA-8	TTGGGTG**F**GTGGGTGGGT	79.7 ± 0.0	3.2
PS-FANA-11	TTGGGTGGGT**F**GGTGGGT	77.2 ± 0.2	0.6
PS-FANA-12	TTGGGTGGGTG**F**GTGGGT	79.3 ± 0.0	2.7
PS-FANA-13	TTGGGTGGGTGG**F**TGGGT	76.6 ± 0.3	0.1
PS-FANA-17	TTGGGTGGGTGGGTGG**F**T	76.3 ± 0.2	−0.2
(3+1) native	TTGGGTTAGGGTTAGGGTTAGGGA	51.4 ± 0.2	
HT-F-3	TT**F**GGTTAGGGTTAGGGTTAGGGA		
HT-F-4	TTG**F**GTTAGGGTTAGGGTTAGGGA	51.6 ± 0.4	0.2
HT-F-5	TTGG**F**TTAGGGTTAGGGTTAGGGA	51.2 ± 0.3	−0.2
HT-F-9	TTGGGTTA**F**GGTTAGGGTTAGGGA		
HT-F-10	TTGGGTTAG**F**GTTAGGGTTAGGGA	48.9 ± 1.2	−2.5
HT-F-11	TTGGGTTAGG**F**TTAGGGTTAGGGA	49.8 ± 0.8	−1.6
HT-F-15	TTGGGTTAGGGTTA**F**GGTTAGGGA		
HT-F-16	TTGGGTTAGGGTTAG**F**GTTAGGGA		
HT-F-17	TTGGGTTAGGGTTAGG**F**TTAGGGA	48.7 ± 0.5	−2.7
HT-F-21	TTGGGTTAGGGTTAGGGTTA**F**GGA		
HT-F-22	TTGGGTTAGGGTTAGGGTTAG**F**GA	49.8 ± 0.8	−1.6
HT-F-23	TTGGGTTAGGGTTAGGGTTAGG**F**A	49.5 ± 0.1	−1.8
HT-FANA-3	TT**F**GGTTAGGGTTAGGGTTAGGGA		
HT-FANA-4	TTG**F**GTTAGGGTTAGGGTTAGGGA	54.5 ± 0.3	3.1
HT-FANA-5	TTGG**F**TTAGGGTTAGGGTTAGGGA	51.9 ± 0.6	0.5
HT-FANA-9	TTGGGTTA**F**GGTTAGGGTTAGGGA		
HT-FANA-10	TTGGGTTAG**F**GTTAGGGTTAGGGA	54.5 ± 0.7	3.2
HT-FANA-11	TTGGGTTAGG**F**TTAGGGTTAGGGA	52.9 ± 0.5	1.5
HT-FANA-15	TTGGGTTAGGGTTA**F**GGTTAGGGA		
HT-FANA-16	TTGGGTTAGGGTTAG**F**GTTAGGGA		
HT-FANA-17	TTGGGTTAGGGTTAGG**F**TTAGGGA	53.8 ± 0.0	2.4
HT-FANA-21	TTGGGTTAGGGTTAGGGTTA**F**GGA		
HT-FANA-22	TTGGGTTAGGGTTAGGGTTAG**F**GA	53.5 ± 0.7	2.2
HT-FANA-23	TTGGGTTAGGGTTAGGGTTAGG**F**A	51.9 ± 0.5	0.6

^a^The ‘HT-series’ denotes sequences modified from the (3+1) G-quadruplex-forming sequence, while the ‘PS-series’ denotes sequences modified from a (4+0) G-quadruplex-forming sequence.

^b^Residues with modified nucleotides are denoted as such: 2′F-RNA-guanosine (**F**) and 2′F-ANA-guanosine (**F**).

^c^Thermal stability data were obtained via UV melting experiments. Salt conditions were (5 mM KCl and 5 mM KPi) for the HT-series and (1 mM KPi) for the PS-series. Data for the HT-series are presented only for sequences that demonstrate a single major conformation in NMR spectra. The uncertainties (±values) indicate the hysteresis between heating and cooling curves. Information regarding thermodynamic parameters ΔH, ΔS and ΔG are presented in Supplementary Table S2.

**Figure 9.**
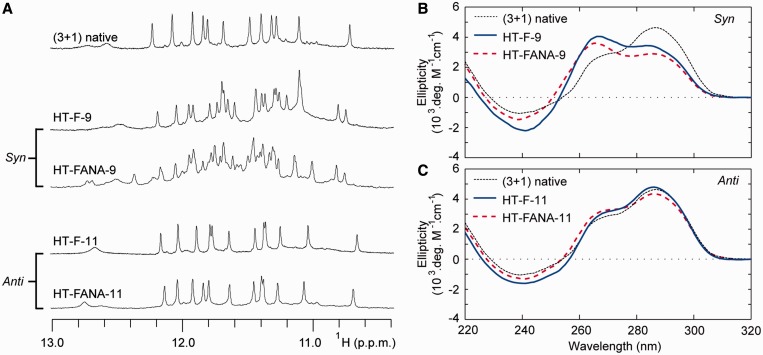
The ‘syn'/‘anti' preference of ^F^G and ^FANA^G substitutions into the (3+1) hybrid G-quadruplex: (**A**) Illustrative NMR spectra of the ‘(3+1) native’ sequence and sequences containing ^F^G or ^FANA^G substitutions to ‘syn’ and ‘anti’ positions. CD spectra are shown for ^F^G and ^FANA^G substitutions to (**B**) ‘syn’ guanine 9 and (**C**) ‘anti’ guanine 11.

Substitutions of ^F^G and ^FANA^G were also made into the (3+1) G-quadruplex scaffold of the HT-series. The substitutions with guanines that adopt a ‘syn’ conformation in the native (3+1) G-quadruplex resulted in multiple sets of imino proton peaks in NMR spectra (Figure 9 and Supplementary Figures S9 and S10). Furthermore, these sequences generally demonstrated notable changes in CD spectra compared with the unmodified sequence, with a decrease in signal at 295 nm and an increase at 260 nm being observed for most sequences (Figure 9 and Supplementary Figures S13 and S14). Alternatively, ^F^G and ^FANA^G were well tolerated when substituted into ‘anti’-position guanines within the (3+1) G-quadruplex. Sequences containing modifications to ‘anti’-position guanines were observed to form a single major conformation in NMR spectra with chemical shift patterns highly similar to that of the unmodified sequence. CD spectra of these sequences were also similar to that of the unmodified sequence, suggesting that modified sequences maintain the same (3+1) G-quadruplex conformation upon substitution of ^F^G or ^FANA^G (Figure 9).

Collectively, these data suggest that modification of ^F^G or ^FANA^G to ‘syn’-position guanines consistently perturbs the folding topology of the (3+1) G-quadruplex and induces a mixture of conformers. The presence of multiple conformations in NMR spectra of ‘syn’-modified sequences is sometimes found to occur without notable change in CD spectra (Supplementary Figures S13 and S14). Such sequences may adopt a mixture of different (3+1) G-quadruplex folding topologies. Previous studies have shown that modifications of ^FANA^G to multiple ‘syn’-positions within a G-quadruplex scaffold can drive conformational changes (25). Works from our laboratory and others have expanded on this to show that rationally placed ^FANA^G modifications can alter structural equilibrium (26,56). In the current work, we demonstrate that a single substitution of either ^F^G or ^FANA^G into any of the ‘syn’-positions tested is powerful enough to perturb the native (3+1) G-quadruplex folding topology. These findings suggest a general destabilizing characteristic of ^F^G or ^FANA^G substitution into ‘syn’-position guanines.

In contrast, ‘anti’-position substitutions of ^F^G and ^FANA^G chemistries are tolerated in both the (3+1) and (4+0) G-quadruplex scaffolds without disrupting the folding topology. The tolerance of these chemistries in all ‘anti’ positions is interesting considering the wide range of structural environments studied in this work. Comparatively, ^LNA^G is less versatile in its ability to be substituted into ‘anti’ conformation guanines. Our work here suggests that, as a general rule, the ^F^G and ^FANA^G chemistries can be universally incorporated into ‘anti’-position guanines in G-quadruplex DNA.

#### Substitutions at anti positions: 2′F-ANA-guanosine generally stabilizes G-quadruplex DNA while 2′F-RNA-guanosine induces mixed effects on stability

**Figure 10.**
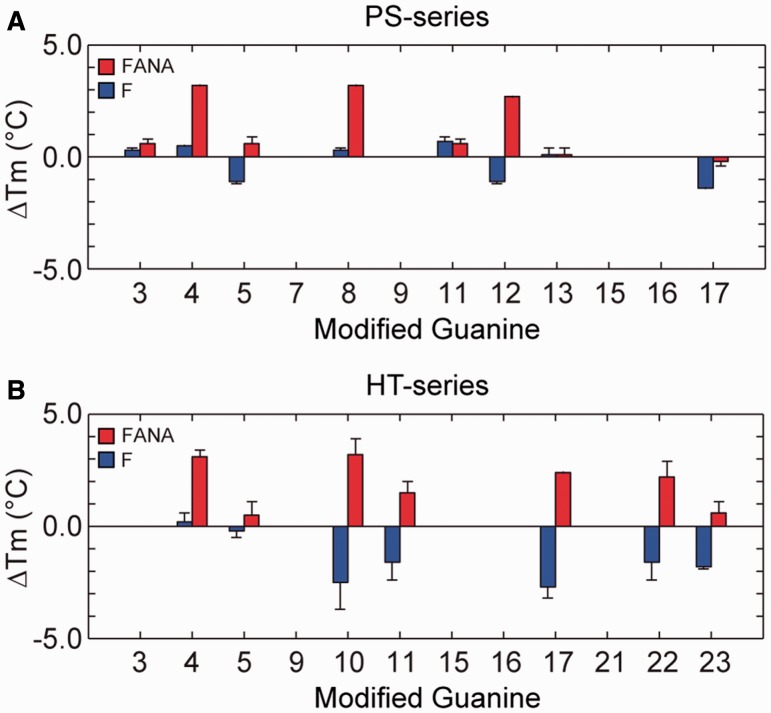
ΔT_m_ for single-position ^F^G and ^FANA^G substitutions to (**A**) select positions in the (4+0) G-quadruplex of the PS-series and (**B**) ‘anti’ positions in the (3+1) G-quadruplex of the HT-series. Error bars indicate the hysteresis between heating and cooling melting curves.

^FANA^G substitutions were generally stabilizing within the (4+0) G-quadruplex scaffold with ΔT_m_ values in the range of −0.2 to +3.2°C and moderately stabilizing in the (3+1) G-quadruplex scaffold with ΔT_m_ values in the range of +0.5 to +3.1°C (Figure 10). Substitutions of ^FANA^G into central tetrad layers in both scaffolds were observed to be particularly stabilizing, with ΔT_m_ values of +2.2 to +3.2°C observed in the (3+1) G-quadruplex HT-series and ΔT_m_ values of +2.7 to +3.2°C observed for the (4+0) G-quadruplex PS-series. On the contrary, sequences containing ^F^G substitutions were generally mildly destabilizing in the HT-series with ΔT_m_ values between −2.7 and +0.2°C and were slightly more favourable in the PS-series with ΔT_m_ values between −1.4 and +0.7°C. No clear tetrad-layer preference is observed for ^F^G substitutions.

The effects of single-position ^F^G and ^FANA^G substitutions on the T_m_ values of G-quadruplexes was determined through thermal denaturing experiments monitored by UV absorption spectroscopy (Figure 10). Samples from the PS-series were analysed in 1 mM KPi, while those from the HT-series were analysed in solution containing 5 mM KCl and 5 mM KPi. Only sequences containing single-position substitutions into anti guanines were analysed as they adopt the same conformation as their parent sequences, a prerequisite to a meaningful quantitative comparison of thermal stability.

The results of our thermal stability studies indicate that ^FANA^G modifications generally lead to a moderate increase in stability. ^F^G substitutions are observed to be tolerated within the two scaffolds, generally decreasing the melting temperature of the (3+1) G-quadruplex while having minor and mixed effects on the stability of the (4+0) G-quadruplex. In all ‘anti’ positions tested over both scaffolds, ^FANA^G is observed to be of equal or greater effectiveness in stabilizing a G-quadruplex structure compared with the ^F^G counterpart. This observation is in agreement with recent work describing ^FANA^G to be a more powerful substituent than ^F^G for driving structural equilibrium (26,56). The different effects of ^F^G and ^FANA^G on the thermal stability of modified G-quadruplexes reported in this work may be attributed to a variety of differing structural features of 2′F-RNA and 2′F-ANA chemistries. Firstly, the sugar pucker in 2′F-ANA adopts a South/East orientation similar to DNA, while 2′F-RNA adopts a North orientation (56,67). These nucleotides also differ in their steric penalties and abilities to form intra-residue hydrogen bonds through substituent fluorine atoms (56,67,68). Interestingly, substitutions of ^FANA^G to guanosines in the central G-tetrad of the three-layered (3+1) and (4+0) G-quadruplexes were found to be most stabilizing compared with other positions. Our current study demonstrates that ^FANA^G are generally better tolerated than ^F^G when substituted into the G-tetrad core of G-quadruplex DNA.

## CONCLUSION

This work examines the effects of systematic single-substitutions of ^LNA^G, ^F^G and ^FANA^G nucleotides into a (3+1) and a (4+0) G-quadruplex scaffold. (I) We discover that modification of ^LNA^G directly upstream of short propeller loops is highly disruptive to G-quadruplex formation. ^LNA^G substitutions are generally tolerated in a manner dependent on the ‘syn’ or ‘anti’ glycosidic conformation of the guanine base. Substitution of ^LNA^G into most ‘anti’ positions leads to a more stable G-quadruplex, while substitution into most ‘syn’ positions generally disrupts the native (3+1) G-quadruplex conformation. However, we identify some noteworthy exceptions to this generalization in the course of our study. (II) A single modification of ^F^G or ^FANA^G to a ‘syn’ guanine in the (3+1) G-quadruplex perturbs this conformation. Alternatively, substitutions into all ‘anti’-positions are well tolerated and do not disrupt the conformation of the (3+1) or the (4+0) G-quadruplexes, suggesting that these nucleotides are universally well-suited for substitution into ‘anti’-position guanine within the G-tetrad core of G-quadruplexes. ^FANA^G is observed to be more stabilizing than ^F^G in both scaffolds, with ^FANA^G substitutions into central G-tetrad guanines being particularly stabilizing. The insight gained from this work will be valuable to the future design of sugar-modified G-quadruplexes for pharmaceutical and engineering applications.

## SUPPLEMENTARY DATA

Supplementary Data are available at NAR Online.

## FUNDING

Singapore Ministry of Education and Nanyang Technological University (to A.T.P.); Nanyang Technological University Undergraduate Research Experience on Campus (URECA) program (to Z.L.). Funding for open access charge: Singapore Ministry of Education.

*Conflict of interest statement*. None declared.

## Supplementary Material

Supplementary Data
