# Supplementary material for: Sugar-modified G-quadruplexes: effects of LNA-, 2′F-RNA– and 2′F-ANA-guanosine chemistries on G-quadruplex structure and stability
Source: Nucleic Acids Res. 2013 Dec 25;42(6):4068–79. doi: 10.1093/nar/gkt1312 (PMC3973314; doi:10.1093/nar/gkt1312)
Supplement: Supplementary Data [file supp_42_6_4068__index.html]

Sugar-modified G-quadruplexes: effects of LNA-, 2′F-RNA– and 2′F-ANA-guanosine chemistries on G-quadruplex structure and stability — Supplementary Data 

# Sugar-modified G-quadruplexes: effects of LNA-, 2′F-RNA– and 2′F-ANA-guanosine chemistries on G-quadruplex structure and stability

## Supplementary Data

files

**Files in this Data Supplement:**

- Supplementary Data - pdf file
