# Supplementary material for: Sugar-modified G-quadruplexes: effects of LNA-, 2′F-RNA– and 2′F-ANA-guanosine chemistries on G-quadruplex structure and stability
Source: Nucleic Acids Res. 2013 Dec 25;42(6):4068–79. doi: 10.1093/nar/gkt1312 (PMC3973314; doi:10.1093/nar/gkt1312)
Supplement: Supplementary Data [file supp_gkt1312_Supplementary_Data.pdf]

Sugar-modified G-quadruplexes: Effects of LNA-, 2'F-RNA- and 2'F-ANA-guanosine chemistries on G-quadruplex structure and stability

Zhe Li[‡], Christopher Jacques Lech[‡] and Anh Tuân Phan*

School of Physical and Mathematical Sciences,

Nanyang Technological University, Singapore 637371

SUPPLEMENTARY DATA

*Corresponding author: phantuan@ntu.edu.sg

[‡]These authors contributed equally to this work.

Running title: LNA, 2'-F- guanosine and 2'-F-ANA-guanosine modifications to G-quadruplexes

Key words: G-quadruplex, LNA-guanosine, 2'-F- guanosine, 2'-F-ANA-guanosine

SUPPORTING INFORMATION CONTENTS

Locked Nucleic Acid modified guanosines

Table S1 – Thermodynamic Parameters of LNA-modified G-quadruplex

Fig S1 – ^{1}H NMR imino proton spectra of (4+0) G-quadruplex-forming PS-series sequences
Fig S2 – ^{1}H NMR imino proton spectra of (3+1) G-quadruplex-forming HT-series sequences
Fig S3 – CD spectra of the (4+0) G-quadruplex-forming PS-series sequences
Fig S4 – CD spectra of the (3+1) G-quadruplex-forming HT-series sequences
Fig S5 – CD melting (4+0) G-quadruplex-forming PS-series sequences
Fig S6 – Fraction Folded CD melting (4+0) G-quadruplex-forming PS-series sequences
Fig S7 – UV melting (3+1) G-quadruplex-forming H-series sequences
Fig S8 – Fraction Folded UV melting (3+1) G-quadruplex-forming H-series sequences

2'-F-guanosine and 2'-F-ANA-guanosine

Table S2 – Thermodynamic Parameters of 2'-F- and2'-F-ANA-modified G-quadruplex

Fig S9 – ^{1}H NMR imino proton spectra of ^{F}G modified (3+1) G-quadruplex-forming sequences
Fig S10 – ^{1}H NMR imino proton spectra of ^{FANA}G modified (3+1) G-quadruplex-forming sequences
Fig S11 – ^{1}H NMR imino proton spectra of ^{F}G modified (4+0) G-quadruplex-forming sequences
Fig S12 – ^{1}H NMR imino proton spectra of ^{FANA}G modified (4+0) G-quadruplex-forming sequences
Fig S13 – CD spectra of ^{F}G modified (3+1) G-quadruplex-forming sequences
Fig S14 – CD spectra of ^{FANA}G modified (3+1) G-quadruplex-forming sequences
Fig S15 – CD spectra of ^{F}G modified (4+0) G-quadruplex-forming sequences
Fig S16 – CD spectra of ^{FANA}G modified (4+0) G-quadruplex-forming sequences
Fig S17 – UV melting of ^{F}G modified (3+1) G-quadruplex-forming sequences
Fig S18 – Fraction Folded UV melting of ^{F}G modified (3+1) G-quadruplex-forming sequences
Fig S19 – UV melting of ^{FANA}G modified (3+1) G-quadruplex-forming sequences
Fig S20 – Fraction Folded UV melting of ^{FANA}G modified (3+1) G-quadruplex-forming sequences
Fig S21 – UV melting of ^{F}G modified (4+0) G-quadruplex-forming sequences
Fig S22 – Fraction Folded UV melting of ^{F}G modified (4+0) G-quadruplex-forming sequences
Fig S23 – UV melting of ^{FANA}G modified (4+0) G-quadruplex-forming sequences
Fig S24 – Fraction Folded UV melting of ^{FANA}G modified (4+0) G-quadruplex-forming sequences

Table S1. Thermodynamic Parameters of LNA-modified G-quadruplex

Name [a]	Sequence (5'→3') [b]	T_m [c] (°C)	ΔH [d] ($kacl•mol^{-1}$)	ΔS [d] ($kacl•mol^{-1}K^{-1}$)	$ΔG_{37°C}$ [d] ($kacl•mol^{-1}$)
(4+0) native	TTGGGTGGGTGGGTGGGT	77.1 ± 0.5	59.6	0.170	6.8 ± 0.0
PS-L3	TT**L**GGTGGGTGGGTGGGT	82.0 ± 0.4	65.4	0.184	8.3 ± 0.0
PS-L4	TTG**L**GTGGGTGGGTGGGT	85.3 ± 0.3	67.1	0.187	9.0 ± 0.1
PS-L5	TTGG**L**TGGGTGGGTGGGT	36.0 ± 0.4	-	-	-
PS-L7	TTGGGT**L**GGTGGGTGGGT	84.3 ± 0.0	77.9	0.218	10.3 ± 0.1
PS-L8	TTGGGTG**L**GTGGGTGGGT	80.2 ± 0.2	69.2	0.196	8.5 ± 0.1
PS-L9	TTGGGTGG**L**TGGGTGGGT	32.2 ± 0.1	-	-	-
PS-L11	TTGGGTGGGT**L**GGTGGGT	84.9 ± 0.1	74.1	0.207	9.9 ± 0.2
PS-L12	TTGGGTGGGTG**L**GTGGGT	79.8 ± 0.1	68.4	0.194	8.3 ± 0.1
PS-L13	TTGGGTGGGTGG**L**TGGGT	31.7 ± 0.5	-	-	-
PS-L15	TTGGGTGGGTGGGT**L**GGT	83.2 ± 0.1	74.3	0.208	9.6 ± 0.9
PS-L16	TTGGGTGGGTGGGTG**L**GT	81.0 ± 0.1	61.8	0.174	7.7 ± 0.2
PS-L17	TTGGGTGGGTGGGTGG**L**T	80.4 ± 0.1	80.5	0.228	9.9 ± 0.8
(3+1) native	TTGGGTTAGGGTTAGGGTTAGGGA	57.4 ± 0.2	61.8	0.187	3.7 ± 0.2
HT-L3	TT**L**GGTTAGGGTTAGGGTTAGGGA	55.4 ± 0.3	60.1	0.183	3.4 ± 0.1
HT-L4	TTG**L**GTTAGGGTTAGGGTTAGGGA	61.6 ± 0.0	63.7	0.190	4.7 ± 0.0
HT-L5	TTGG**L**TTAGGGTTAGGGTTAGGGA	56.8 ± 0.4	55.1	0.167	3.3 ± 0.1
HT-L9	TTGGGTTA**L**GGTTAGGGTTAGGGA	-	-	-	-
HT-L10	TTGGGTTAG**L**GTTAGGGTTAGGGA	59.3 ± 0.5	62.9	0.189	4.2 ± 0.1
HT-L11	TTGGGTTAGG**L**TTAGGGTTAGGGA	58.3 ± 0.3	62.6	0.189	4.0 ± 0.1
HT-L15	TTGGGTTAGGGTTA**L**GGTTAGGGA	-	-	-	-
HT-L16	TTGGGTTAGGGTTAG**L**GTTAGGGA	-	-	-	-
HT-L17	TTGGGTTAGGGTTAGG**L**TTAGGGA	55.1 ± 0.4	47.5	0.145	2.6 ± 0.1
HT-L21	TTGGGTTAGGGTTAGGGTTA**L**GGA	-	-	-	-
HT-L22	TTGGGTTAGGGTTAGGGTTAG**L**GA	-	-	-	-
HT-L23 [e]	TTGGGTTAGGGTTAGGGTTAGG**L**A	60.5 ± 0.1	52.8	0.158	3.7 ± 0.0

[a] The "HT-series" denotes sequences modified from the (3+1) G-quadruplex forming sequence, while the "PS-series" denotes sequences modified from a (4+0) G-quadruplex forming sequence.
[b] Residues with LNA-modified guanosine are denoted as (**L**)
[c] Thermal stability data was obtained via UV melting (HT-series) and CD melting (PS-series) experiments. Salt conditions were (20 mM KPi) for the HT-series and (1.1 mM KPi) for the PS-series. Thermal stability data for the HT-series is presented for sequences which demonstrate a single species in NMR spectra. The uncertainties (± values) indicate the hysteresis between heating and cooling curves.
[d] The values of ΔH and ΔS were deduced from a slope analysis of fraction folded curves assuming a G-quadruplex to single strand transition (unfolding event). $ΔG_{37°C}$ was calculated from the relation ΔG(T) = ΔH-TΔS where T=310°K. The uncertainties (± values) indicate the difference between $ΔG_{37°C}$ calculated from heating and cooling curves.
[e] Sequence contains a small secondary melting transition at the low temperature range.

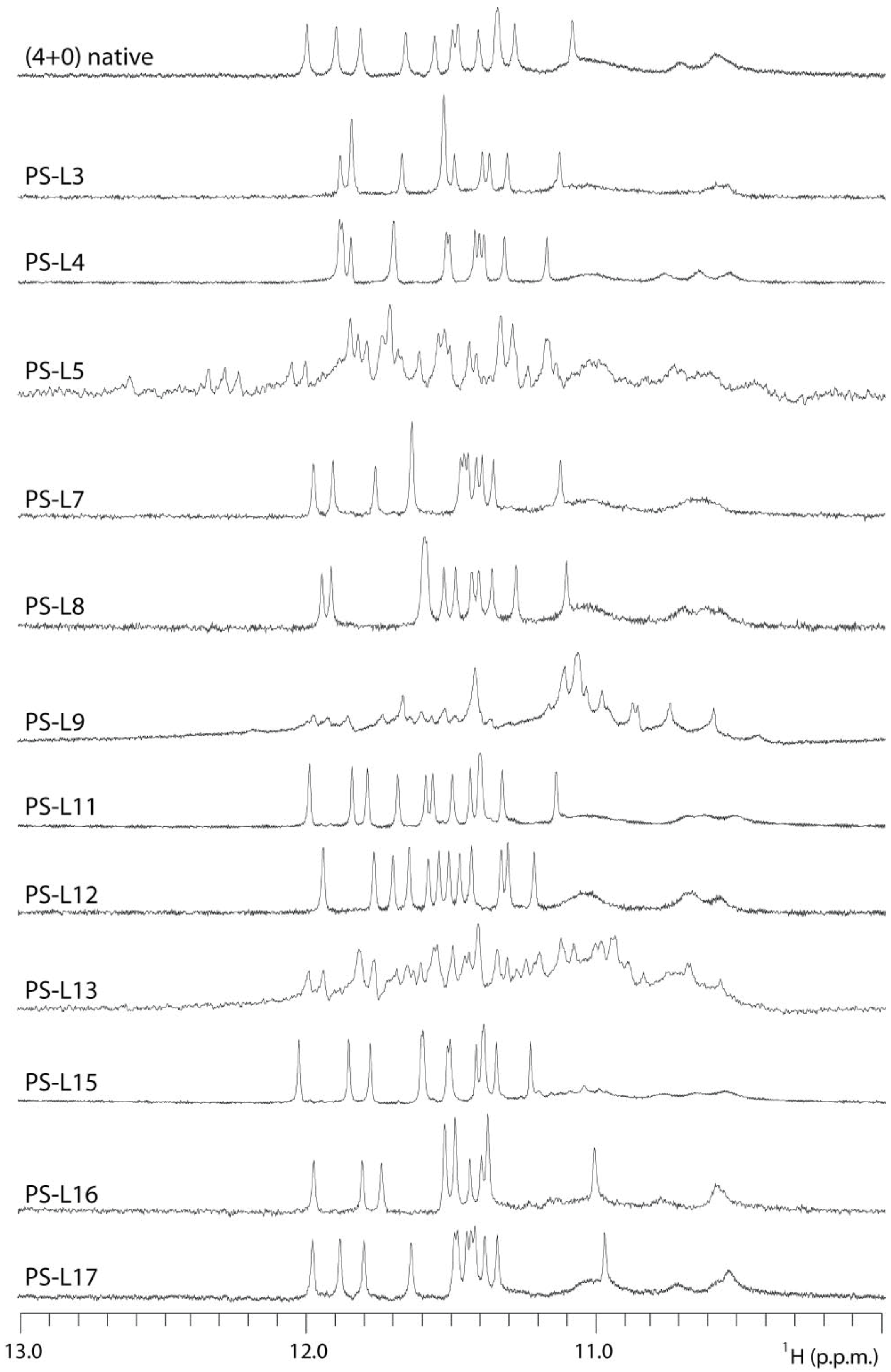

Figure S1 – ^{1}H NMR imino proton spectra of (4+0) G-quadruplex-forming PS-Series sequences.

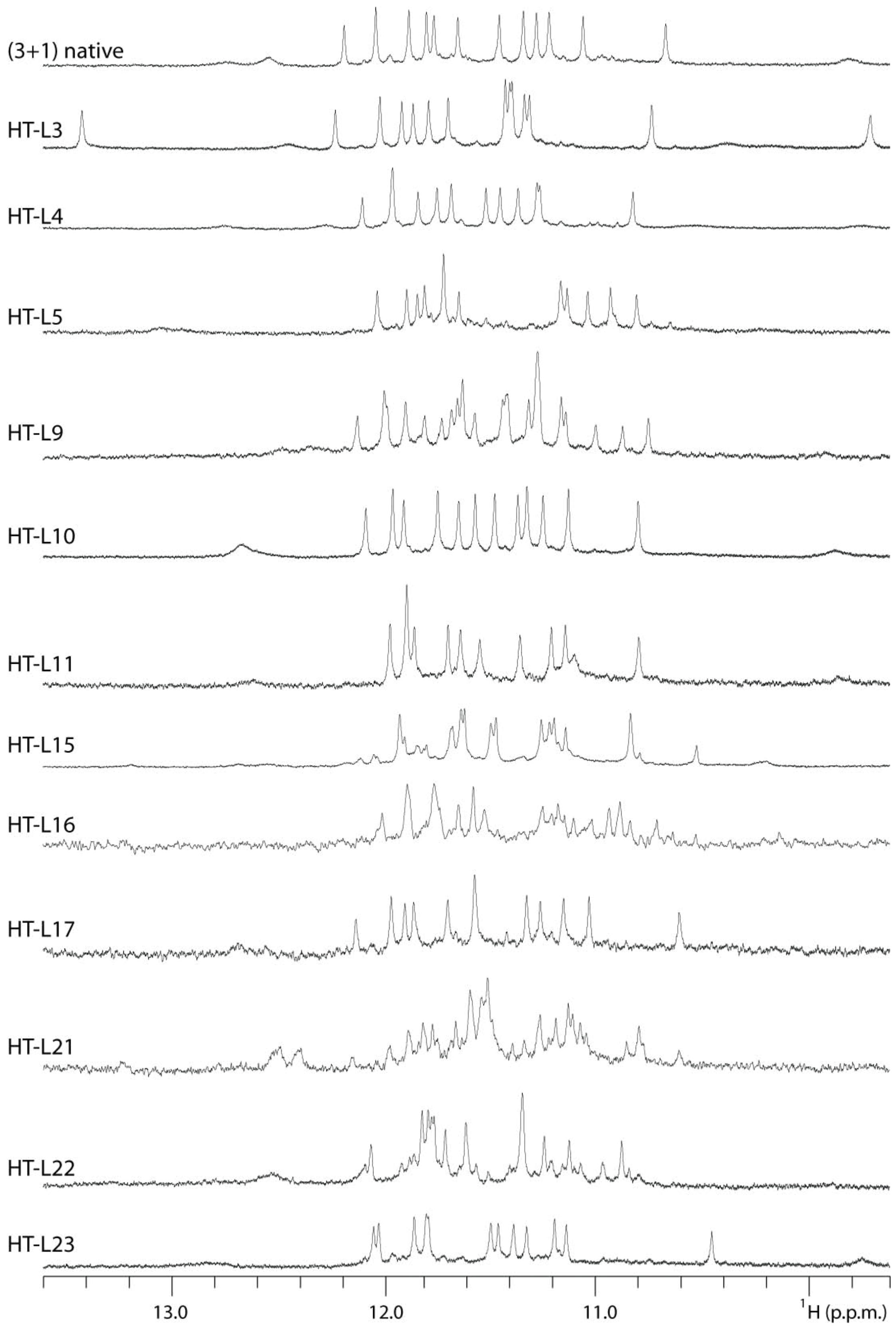

Figure S2 – ^{1}H NMR imino proton spectra of (3+1) G-quadruplex-forming HT-Series sequences.

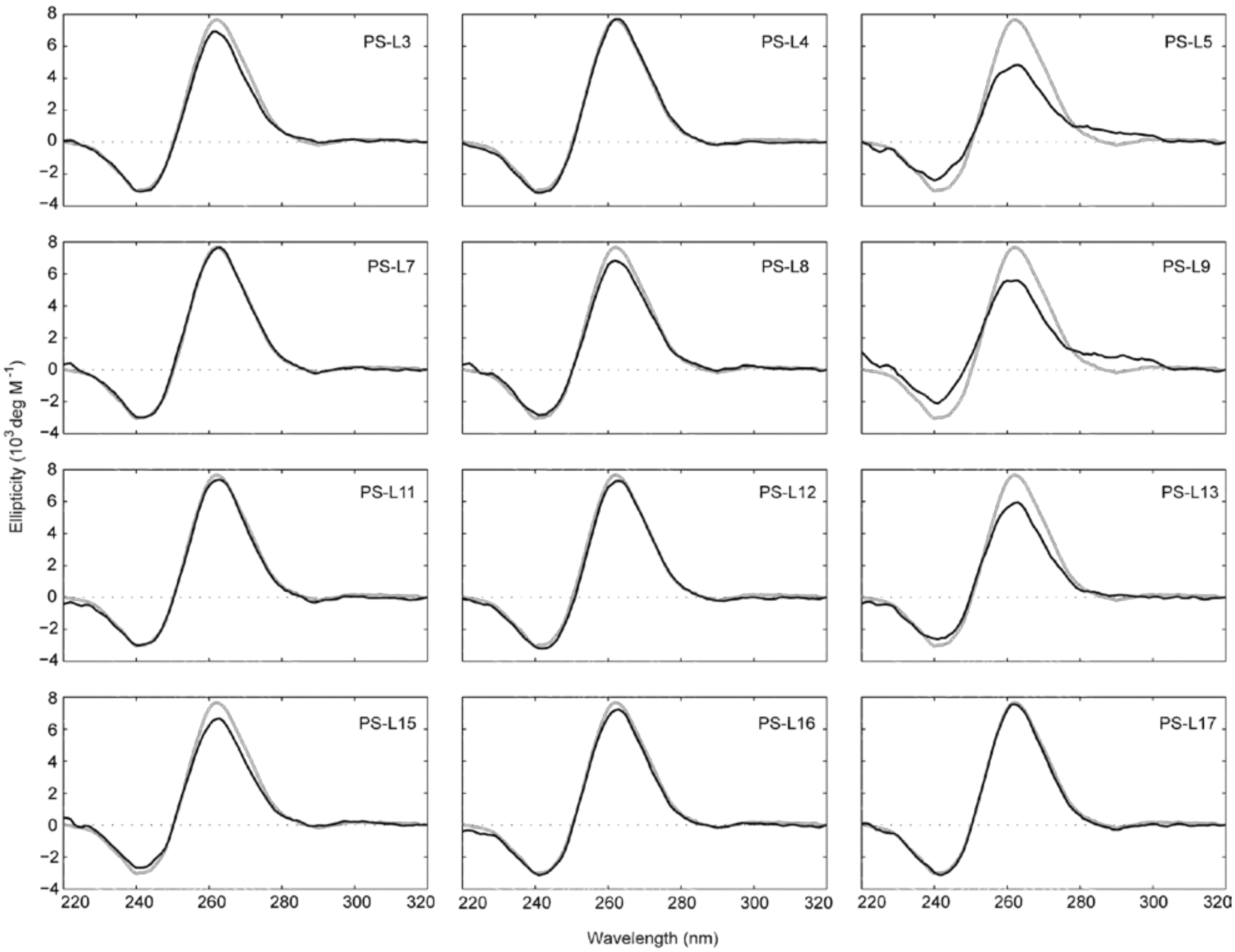

Fig S3 – CD spectra of the (4+0) G-quadruplex-forming PS-series sequences: CD spectra of modified sequences (black) and the (4+0) G-quadruplex native sequence (grey) are shown.

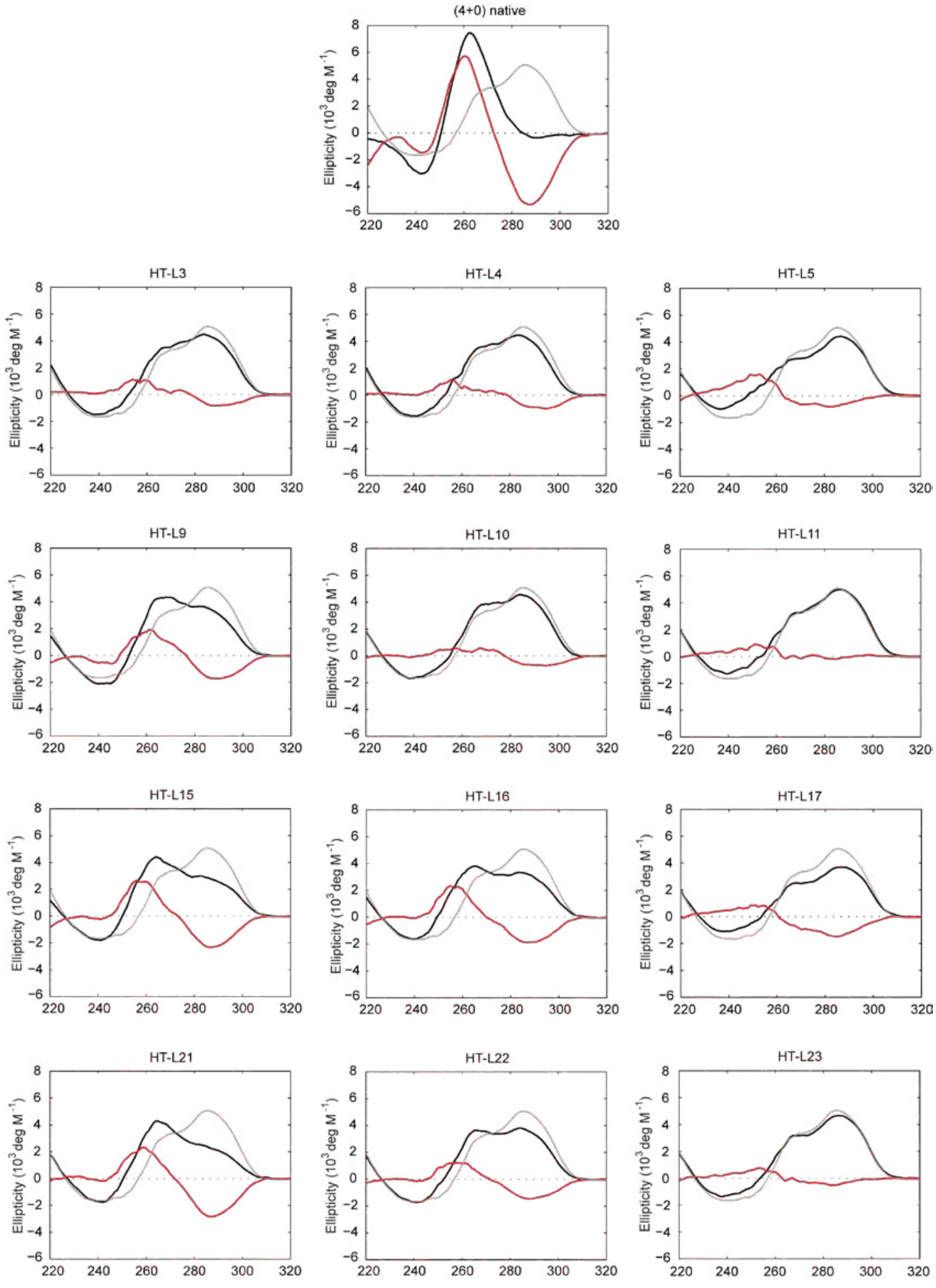

Figure S4 – CD spectra of the (3+1) G-quadruplex-forming HT-series sequences: CD Spectra of modified sequences (black) and the (3+1) G-quadruplex native sequence (grey) are shown. CD Difference spectra (red) are determined by subtracting the (3+1) G-quadruplex native from the modified spectra. The CD spectrum and CD difference spectrum of the (4+0) G-quadruplex native sequence is shown for reference (top).

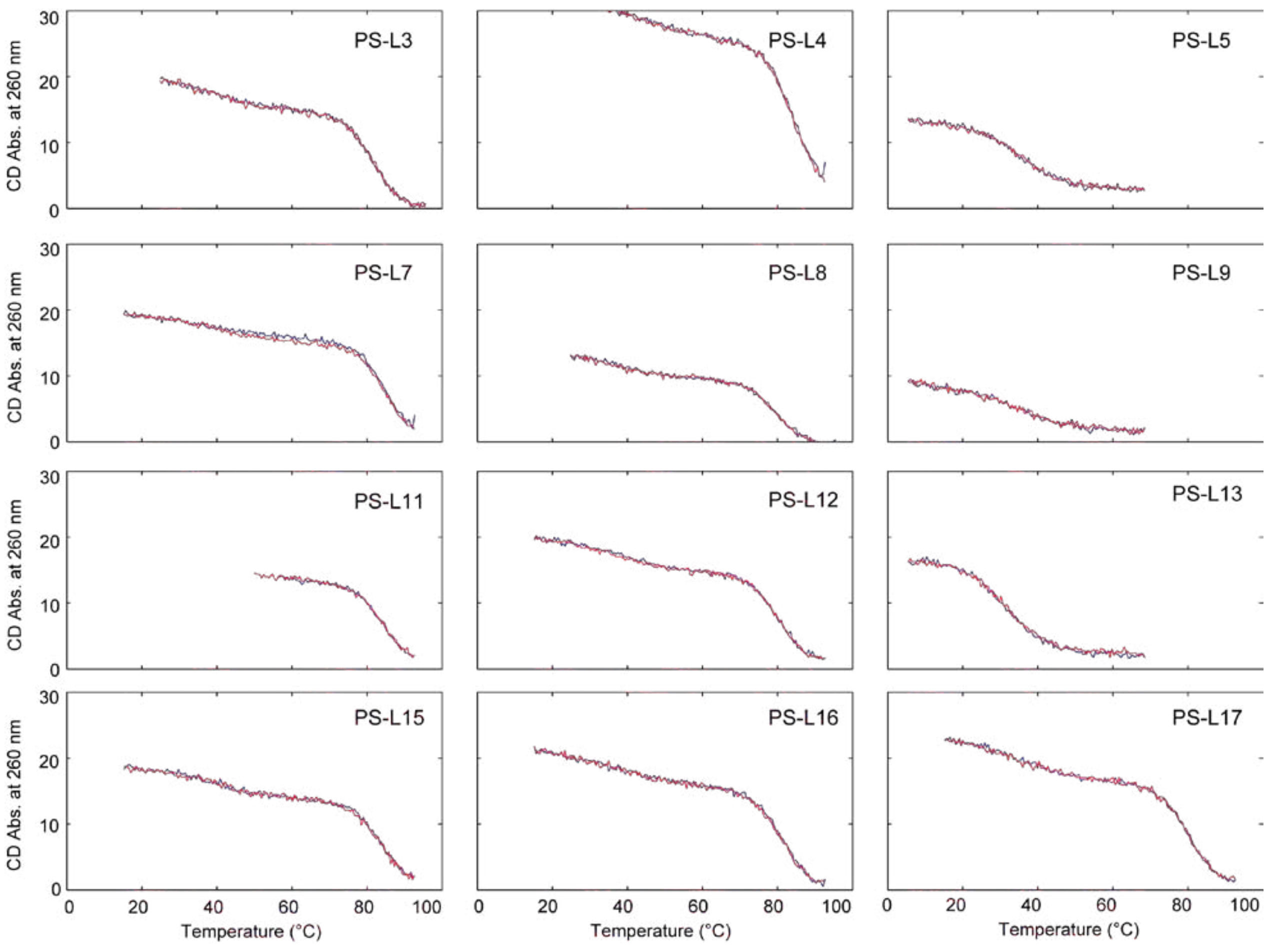

Fig S5 –CD Melting (4+0) G-quadruplex-forming PS-series sequences. Cooling curve is shown in blue, heating in red.

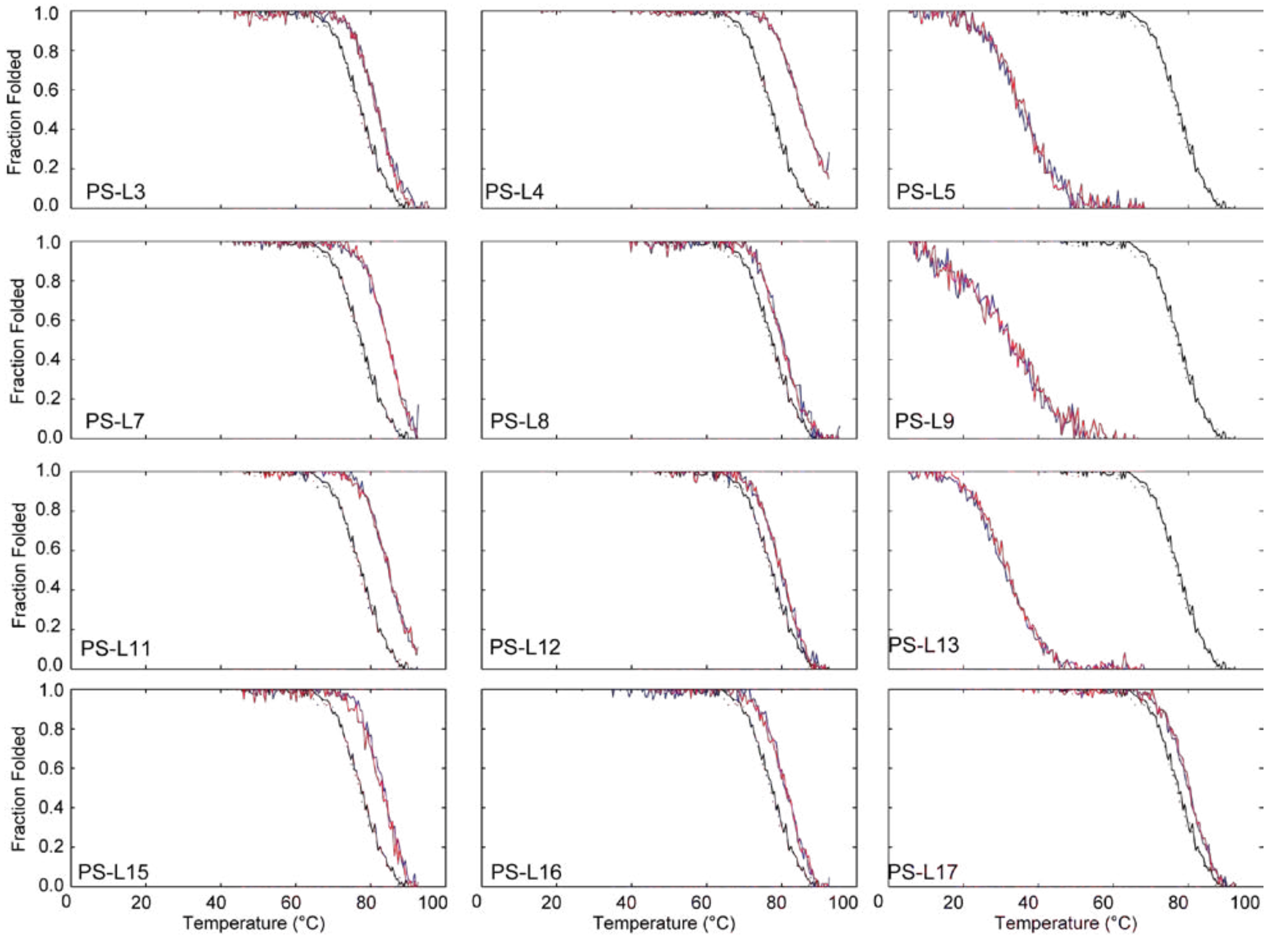

Fig S6 – Fraction folded CD Melting (4+0) G-quadruplex-forming PS-series sequences. Cooling curve is shown in blue, heating in red. The melting curve of the (4+0) G-quadruplex native sequence is shown in black.

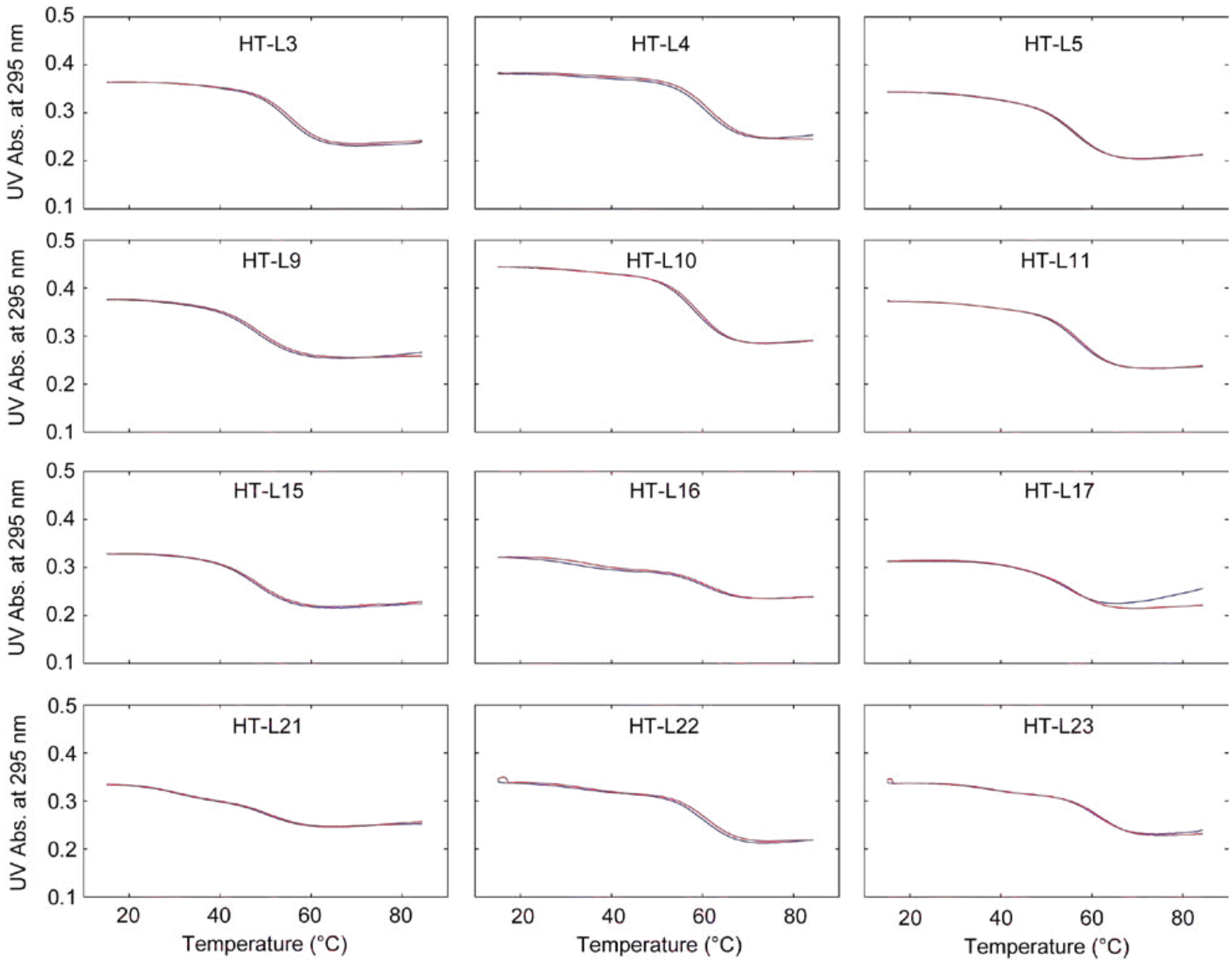

Fig S7 – UV Melting (3+1) G-quadruplex-forming HT-Series sequences. Cooling curve is shown in blue, heating in red.

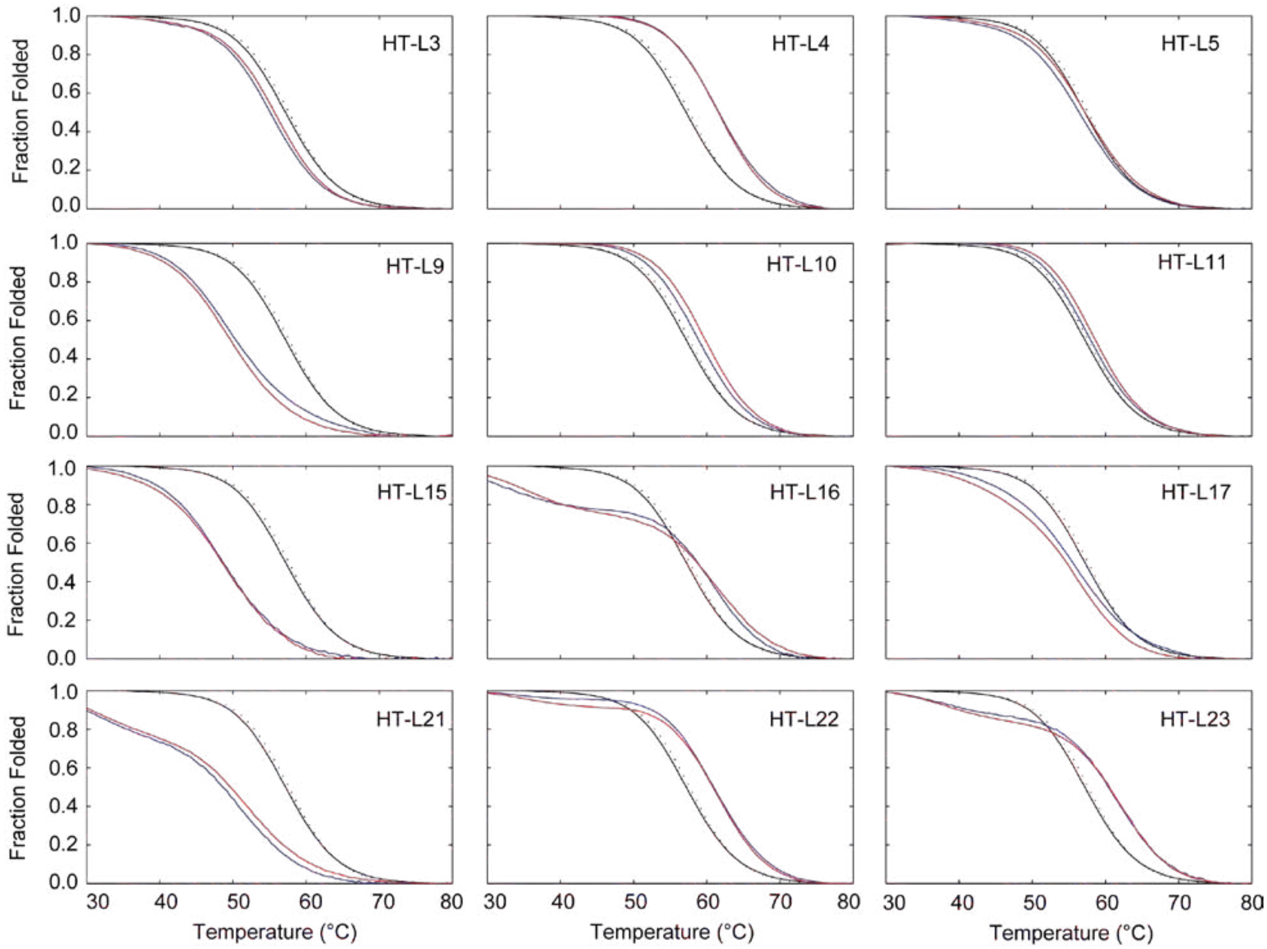

Fig S8 – Fraction folded CD melting (3+1) G-quadruplex-forming HT-Series sequences. Cooling curve is shown in blue, heating in red. The melting curve of the (3+1) G-quadruplex native sequence is shown in black.

Table S2. Thermodynamic Parameters of 2’-F- and2’-F-ANA-modified G-quadruplex

Name [a]	Sequence (5’→3’) [b]	T_m [c] (°C)	ΔH [d] (kacl•mol^{-1})	ΔS [d] (kacl•mol^{-1}K^{-1})	$\Delta G_{37°C}$ [d] (kacl•mol^{-1})
(4+0) native	TTGGGTGGGTGGGTGGGT	76.5 ± 0.4	68.9	0.197	7.8 ± 0.1
PS-F-3	TT**F**GGTGGGTGGGTGGGT	76.8 ± 0.1	70.6	0.202	8.0 ± 0.3
PS-F-4	TTG**F**GTGGGTGGGTGGGT	77.0 ± 0.0	69.2	0.198	7.9 ± 0.0
PS-F-5	TTGG**F**TGGGTGGGTGGGT	75.4 ± 0.1	66.6	0.191	7.3 ± 0.3
PS-F-8	TTGGGTG**F**GTGGGTGGGT	76.9 ± 0.1	67.5	0.193	7.7 ± 0.1
PS-F-11	TTGGGTGGGT**F**GGTGGGT	77.2 ± 0.2	74.2	0.212	8.5 ± 0.0
PS-F-12	TTGGGTGGGTG**F**GTGGGT	75.4 ± 0.1	67.9	0.195	7.5 ± 0.0
PS-F-13	TTGGGTGGGTGG**F**TGGGT	76.6 ± 0.3	66.1	0.189	7.5 ± 0.2
PS-F-17	TTGGGTGGGTGGGTGG**F**T	75.2 ± 0.0	72.5	0.208	7.9 ± 0.2
PS-FANA-3	TT**F**GGTGGGTGGGTGGGT	77.1 ± 0.2	67.5	0.193	7.7 ± 0.2
PS-FANA-4	TTG**F**GTGGGTGGGTGGGT	79.7 ± 0.0	68.2	0.193	8.3 ± 0.1
PS-FANA-5	TTGG**F**TGGGTGGGTGGGT	77.2 ± 0.3	68.8	0.196	7.9 ± 0.2
PS-FANA-8	TTGGGTG**F**GTGGGTGGGT	79.7 ± 0.3	64.5	0.183	7.8 ± 0.2
PS-FANA-11	TTGGGTGGGT**F**GGTGGGT	77.2 ± 0.2	69.8	0.199	8.0 ± 0.0
PS-FANA-12	TTGGGTGGGTG**F**GTGGGT	79.3 ± 0.0	72.4	0.205	8.7 ± 0.2
PS-FANA-13	TTGGGTGGGTGG**F**TGGGT	76.6 ± 0.3	66.9	0.191	7.6 ± 0.3
PS-FANA-17	TTGGGTGGGTGGGTGG**F**T	76.3 ± 0.2	68.0	0.195	7.6 ± 0.1
(3+1) native	TTGGGTTAGGGTTAGGGTTAGGGA	51.4 ± 0.2	61.7	0.190	2.7 ± 0.1
HT-F-3	TT**F**GGTTAGGGTTAGGGTTAGGGA	-	-	-	-
HT-F-4	TTG**F**GTTAGGGTTAGGGTTAGGGA	51.6 ± 0.4	63.1	0.194	2.8 ± 0.1
HT-F-5	TTGG**F**TTAGGGTTAGGGTTAGGGA	51.2 ± 0.3	62.9	0.194	2.8 ± 0.2
HT-F-9	TTGGGTTA**F**GGTTAGGGTTAGGGA	-	-	-	-
HT-F-10	TTGGGTTAG**F**GTTAGGGTTAGGGA	48.9 ± 1.2	60.4	0.187	2.2 ± 0.4
HT-F-11	TTGGGTTAGG**F**TTAGGGTTAGGGA	49.8 ± 0.8	63.8	0.197	2.5 ± 0.3
HT-F-15	TTGGGTTAGGGTTA**F**GGTTAGGGA	-	-	-	-
HT-F-16	TTGGGTTAGGGTTAG**F**GTTAGGGA	-	-	-	-
HT-F-17	TTGGGTTAGGGTTAGG**F**TTAGGGA	48.7 ± 0.5	59.0	0.183	2.2 ± 0.3
HT-F-21	TTGGGTTAGGGTTAGGGTTA**F**GGA	-	-	-	-
HT-F-22	TTGGGTTAGGGTTAGGGTTAG**F**GA	49.8 ± 0.8	66.5	0.206	2.7 ± 0.4
HT-F-23	TTGGGTTAGGGTTAGGGTTAGG**F**A	49.5 ± 0.1	62.3	0.193	2.4 ± 0.2
HT-FANA-3	TT**F**GGTTAGGGTTAGGGTTAGGGA	-	-	-	-
HT-FANA-4	TTG**F**GTTAGGGTTAGGGTTAGGGA	54.5 ± 0.3	66.9	0.204	3.6 ± 0.2
HT-FANA-5	TTGG**F**TTAGGGTTAGGGTTAGGGA	51.9 ± 0.6	61.3	0.189	2.8 ± 0.3
HT-FANA-9	TTGGGTTA**F**GGTTAGGGTTAGGGA	-	-	-	-
HT-FANA-10	TTGGGTTAG**F**GTTAGGGTTAGGGA	54.5 ± 0.7	65.5	0.200	3.5 ± 0.2
HT-FANA-11	TTGGGTTAGG**F**TTAGGGTTAGGGA	52.9 ± 0.5	62.2	0.191	3.0 ± 0.2
HT-FANA-15	TTGGGTTAGGGTTA**F**GGTTAGGGA	-	-	-	-
HT-FANA-16	TTGGGTTAGGGTTAG**F**GTTAGGGA	-	-	-	-
HT-FANA-17	TTGGGTTAGGGTTAGG**F**TTAGGGA	53.8 ± 0.0	60.6	0.185	3.1 ± 0.3
HT-FANA-21	TTGGGTTAGGGTTAGGGTTA**F**GGA	-	-	-	-
HT-FANA-22	TTGGGTTAGGGTTAGGGTTAG**F**GA	53.5 ± 0.7	61.8	0.189	3.1 ± 0.1
HT-FANA-23	TTGGGTTAGGGTTAGGGTTAGG**F**A	51.9 ± 0.5	66.3	0.204	3.1 ± 0.2

[a] The “HT-series” denotes sequences modified from the (3+1) G-quadruplex forming sequence, while the “PS-series” denotes sequences modified from a (4+0) G-quadruplex forming sequence.
[b] Residues with modified nucleotides are denoted as such: 2’F-guanosine (**F**) and 2’F-ANA-guanosine (**F**).
[c] Thermal stability data was obtained via UV melting experiments. Salt conditions were (5 mM KCl and 5 mM KPi) for the HT-series and (1 mM KPi) for the PS-series. Data for the HT-series is presented for sequences which demonstrate a single species in NMR spectra. The uncertainties (± values) indicate the hysteresis between heating and cooling curves.
[d] The values of ΔH and ΔS were deduced from a slope analysis of fraction folded curves assuming a G-quadruplex to single strand transition (unfolding event). $\Delta G_{37°C}$ was calculated from the relation ΔG(T) = ΔH-TΔS where T=310°K. The uncertainties (± values) indicate the difference between $\Delta G_{37°C}$ calculated from heating and cooling curves.

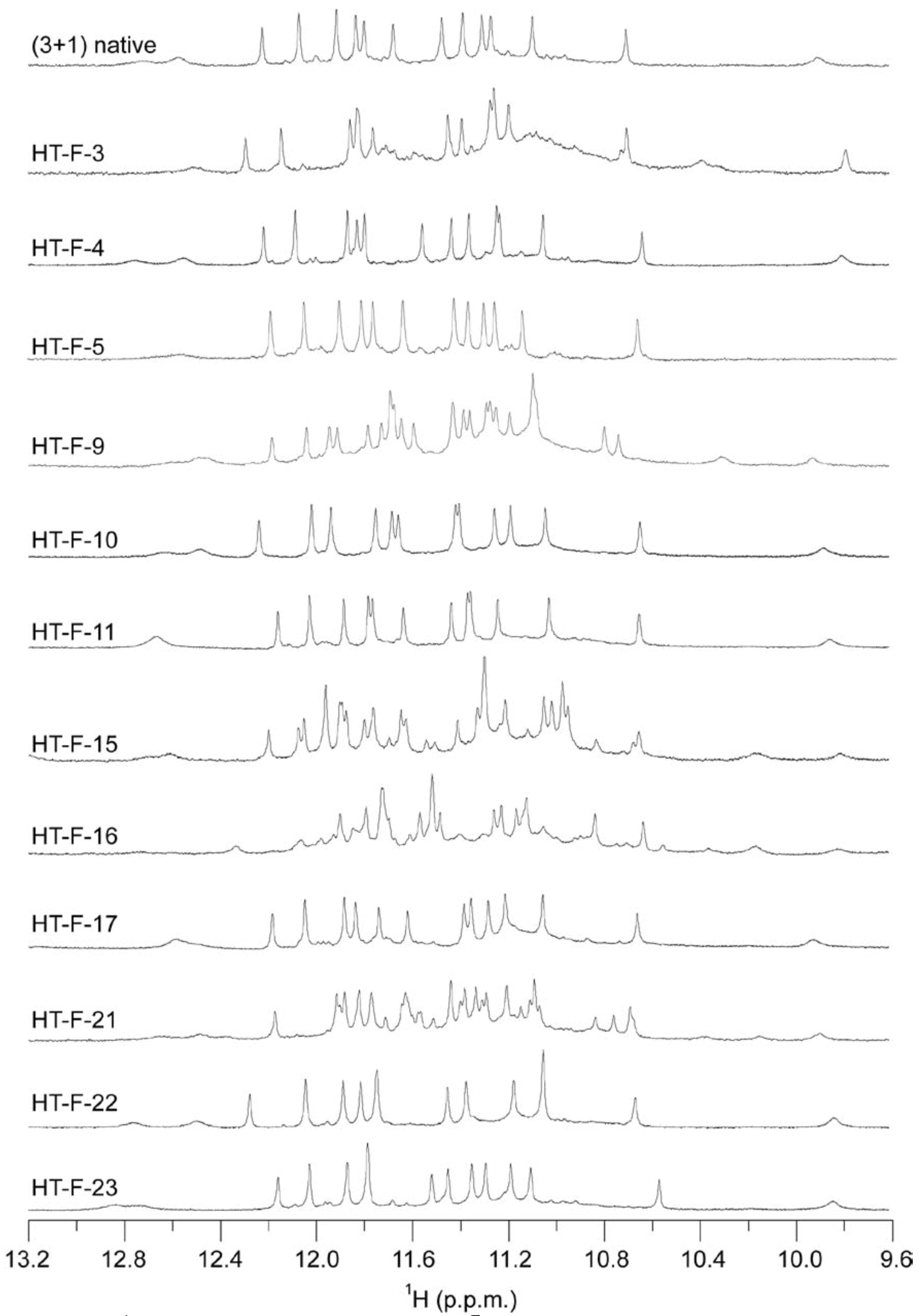

Figure S9: ^{1}H NMR imino proton spectra of ^{F}G modified (3+1) G-quadruplex-forming sequences

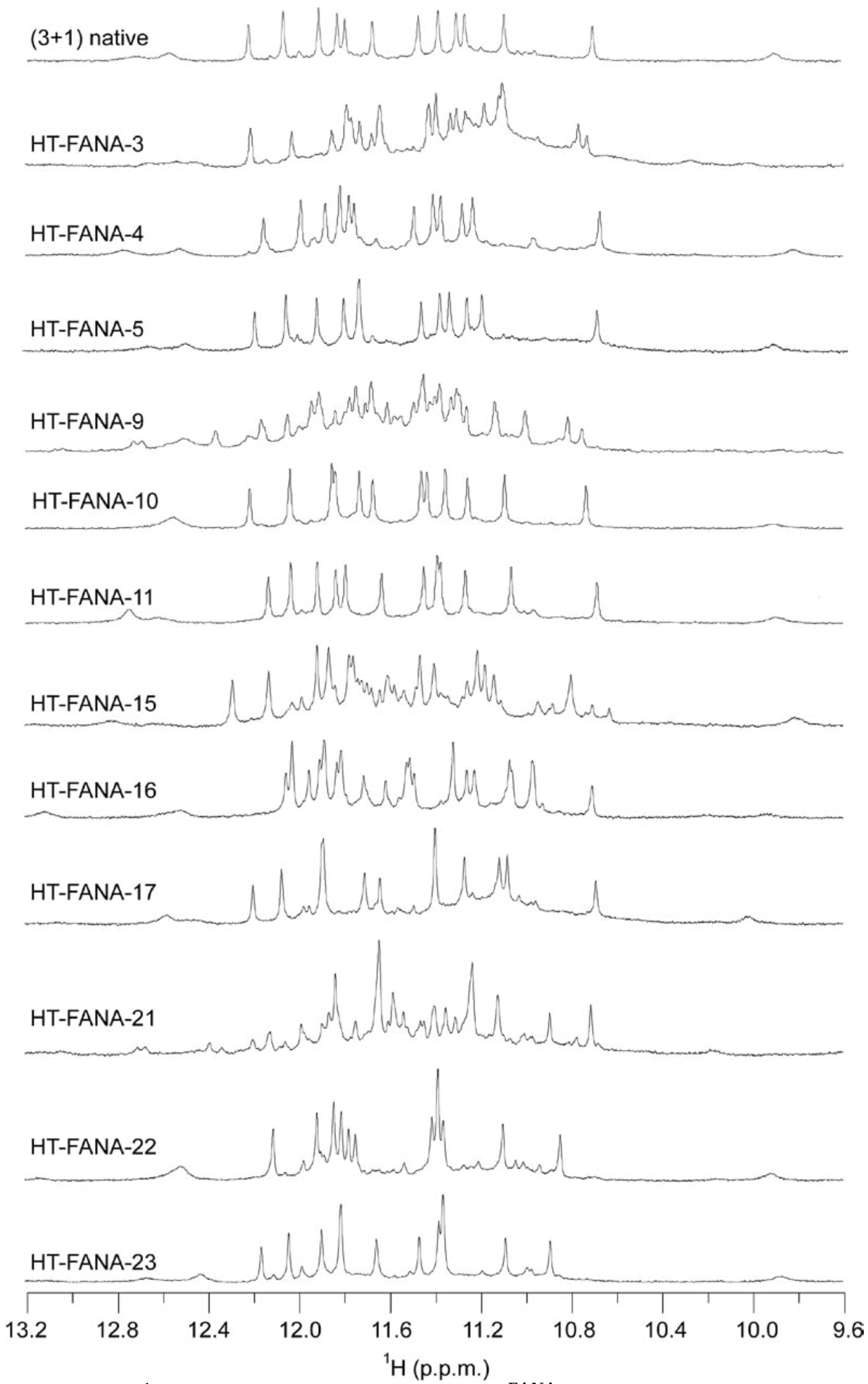

Figure S10: ^{1}H NMR imino proton spectra of ^{FANA}G modified (3+1) G-quadruplex-forming sequences

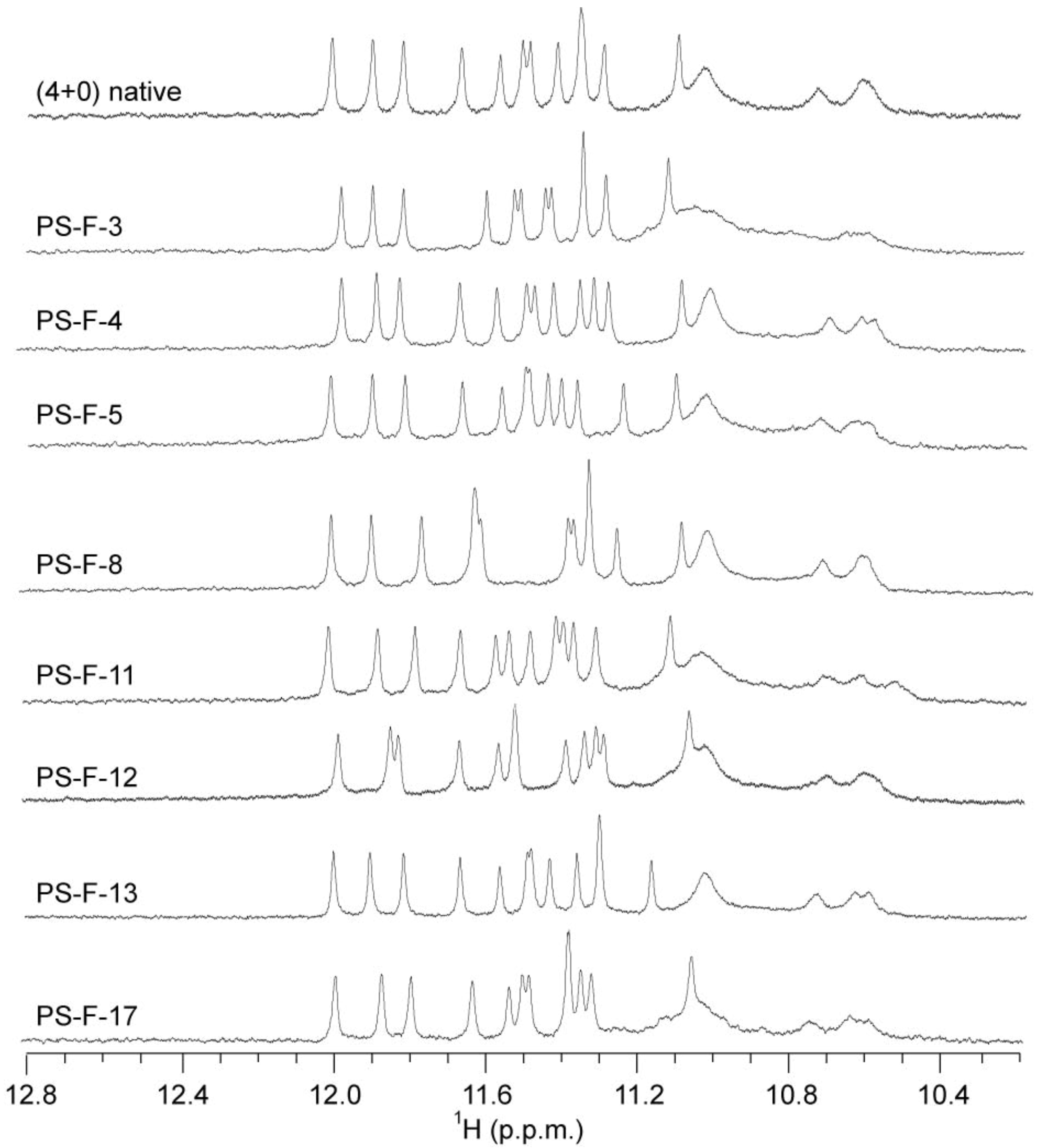

Figure S11: ^{1}H NMR imino proton spectra of ^{F}G modified (4+0) G-quadruplex-forming sequences

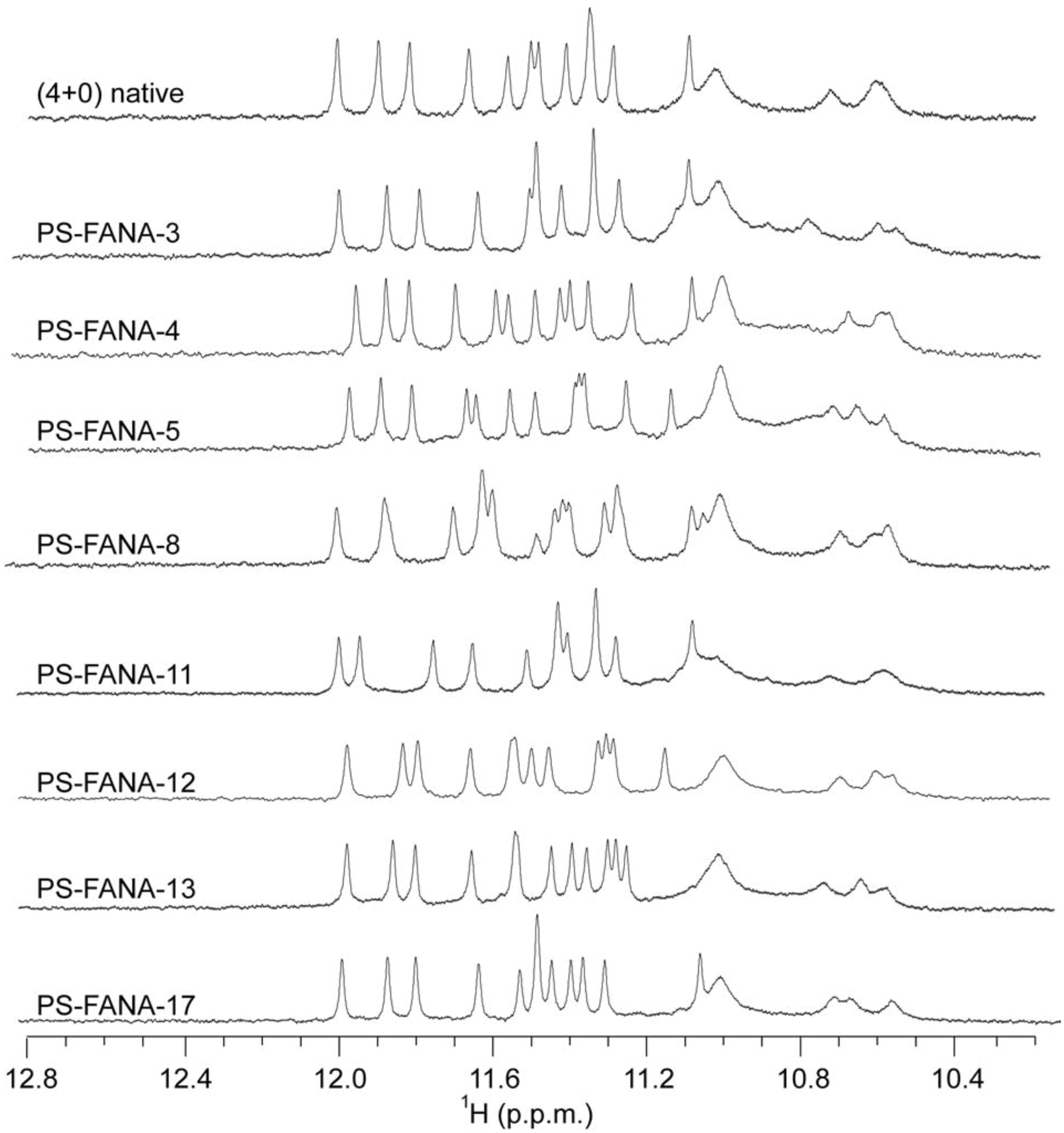

Figure S12: ^{1}H NMR imino proton spectra of ^{FANA}G modified (4+0) G-quadruplex-forming sequences

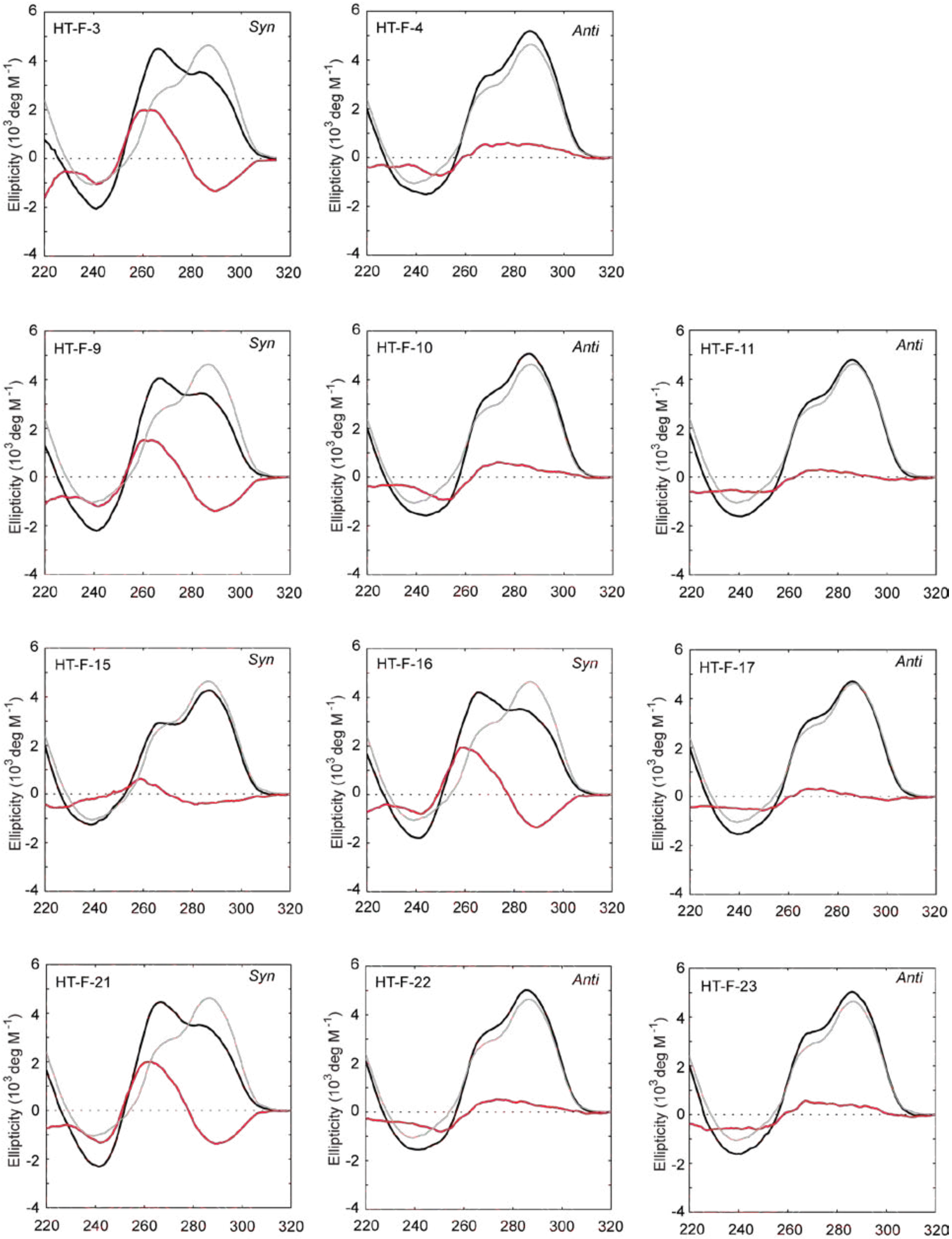

Figure S13: CD spectra (black) of ^{F}G-modified HT-series. Native (3+1) G-quadruplex is shown as a reference (grey). Difference spectra (red) compare the modified sequence to the native one.

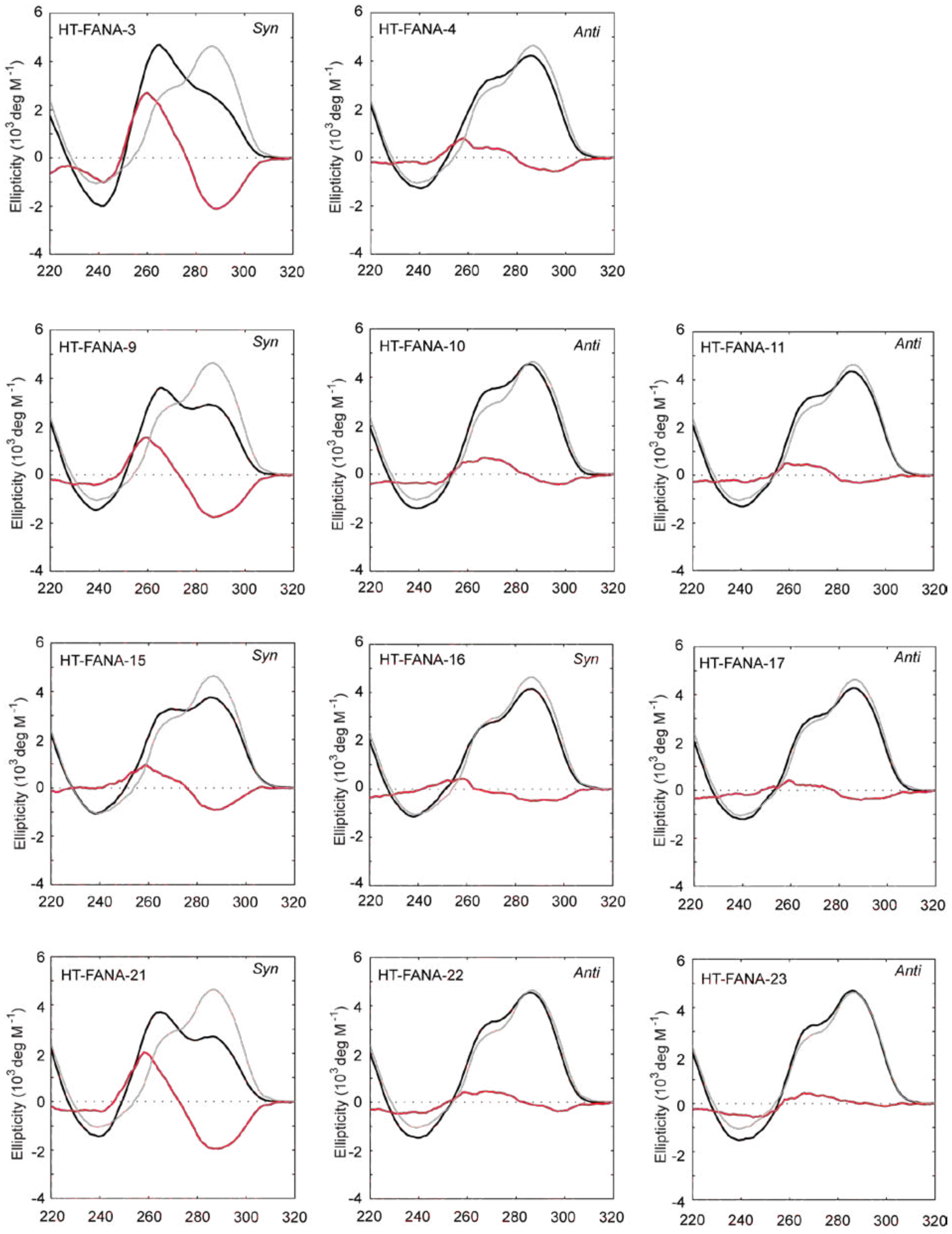

Figure S14: CD spectra (black) of ^{FANA}G-modified HT-series. Native (3+1) G-quadruplex is shown as a reference (grey). Difference spectra (red) compare the modified sequence to the native one.

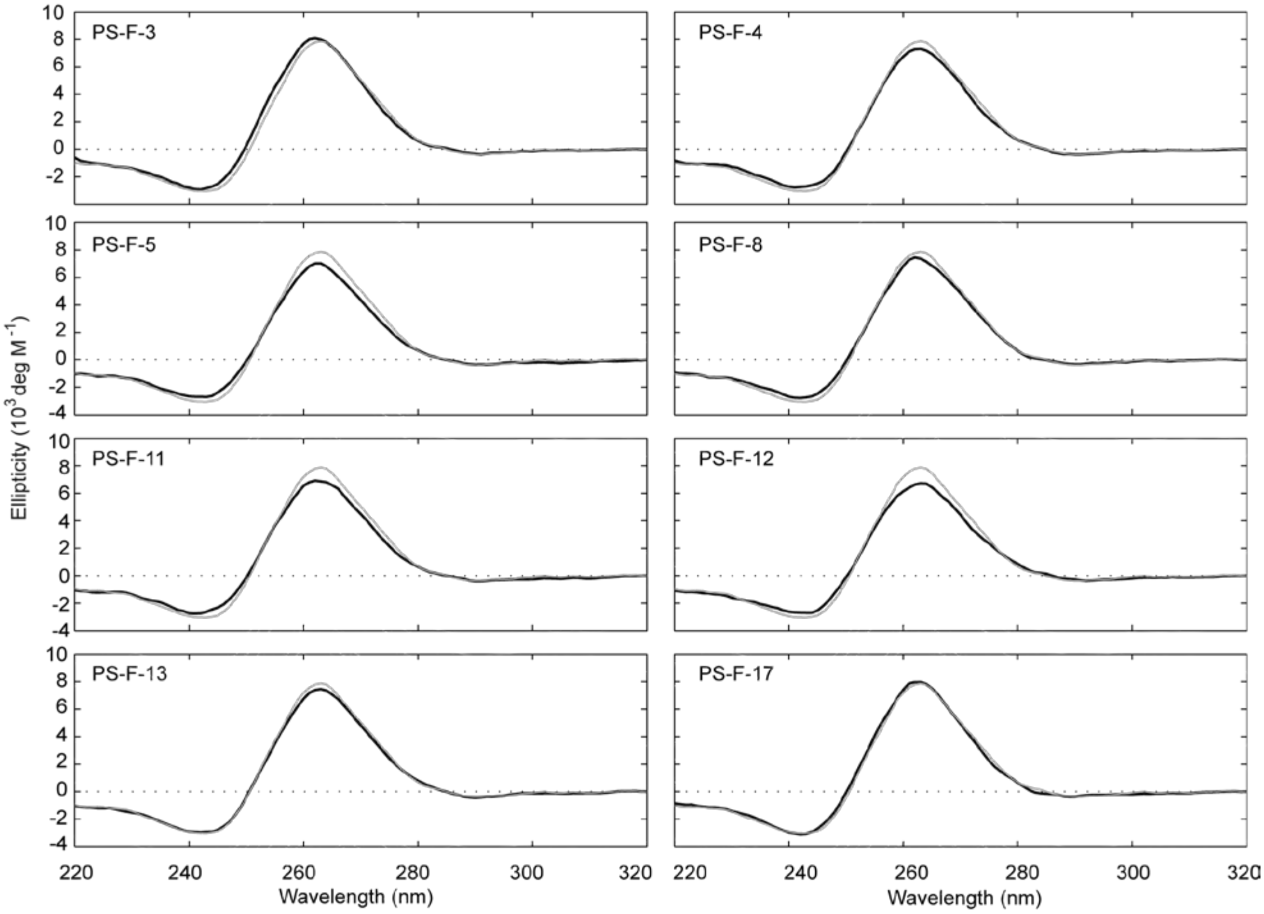

Figure S15: CD spectra (black) of ^{F}G-modified PS-series. Native (4+0) G-quadruplex is shown as a reference (grey).

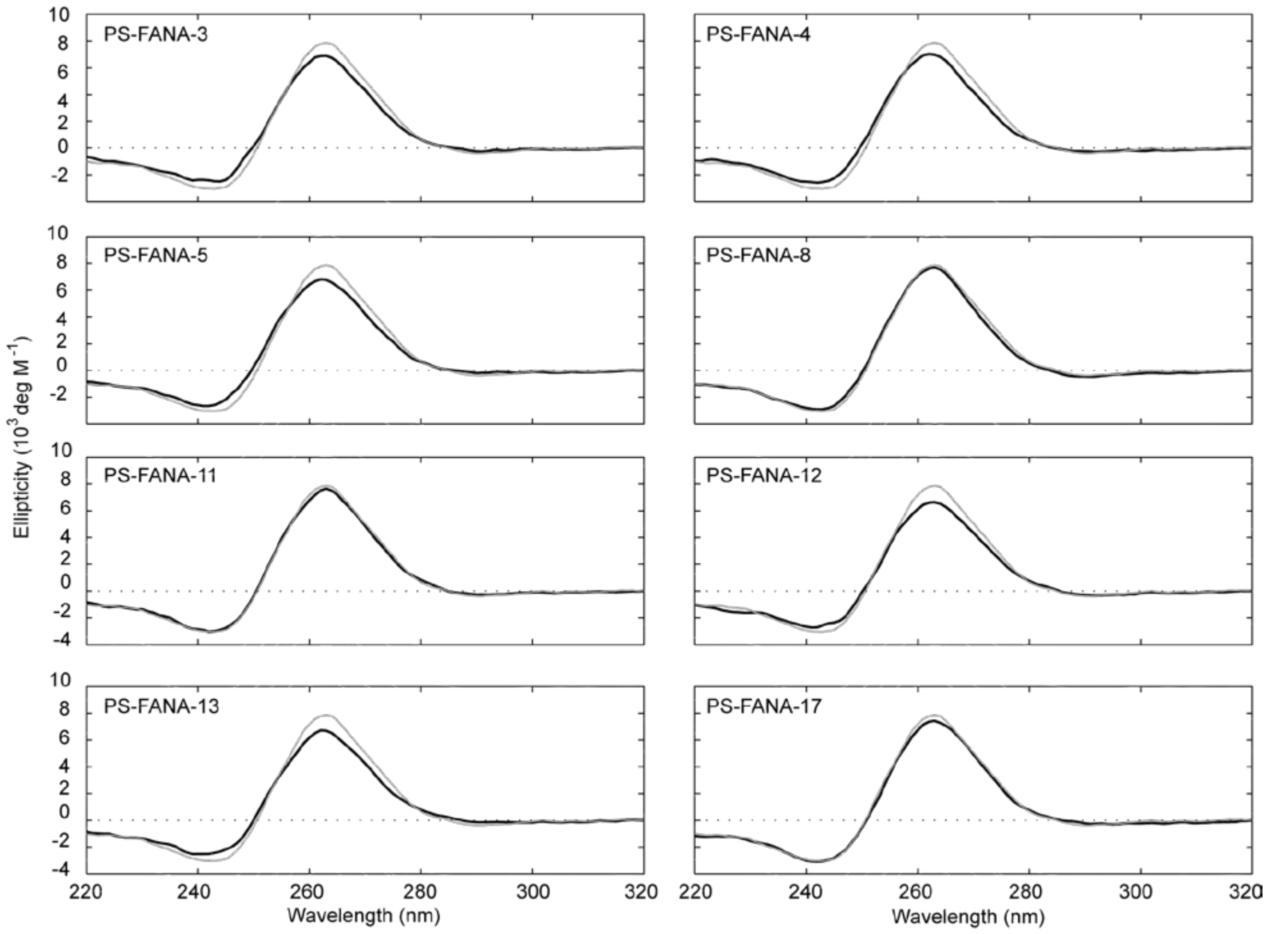

Figure S16: CD spectra (black) of ^{FANA}G-modified PS-series. Native (4+0) G-quadruplex is shown as a reference (grey).

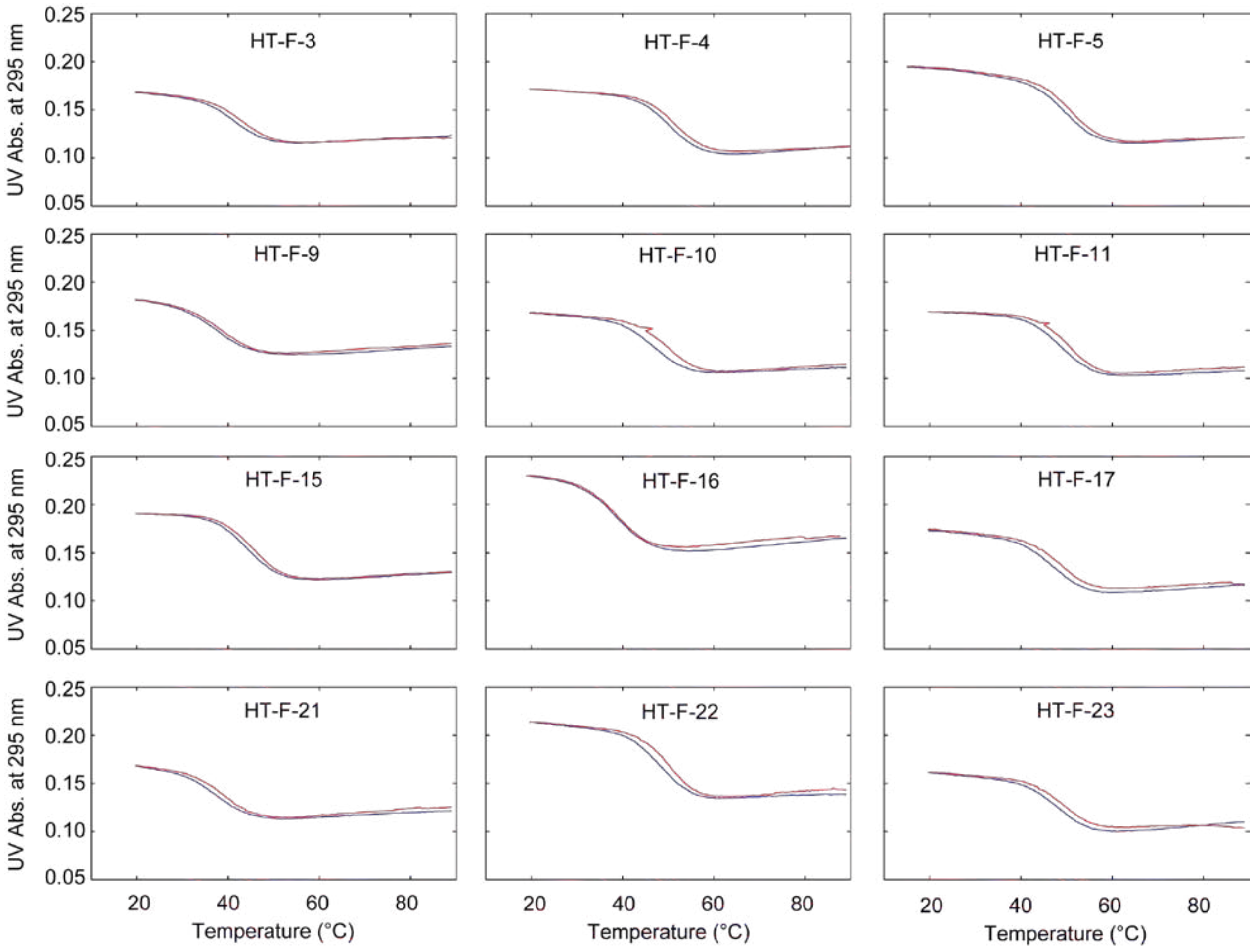

Figure S17: UV absorbance spectra at 295 nm of thermal denaturing experiments of ^{F}G-modified HT-series sequences. Both heating (red) and cooling (blue) curves are shown.

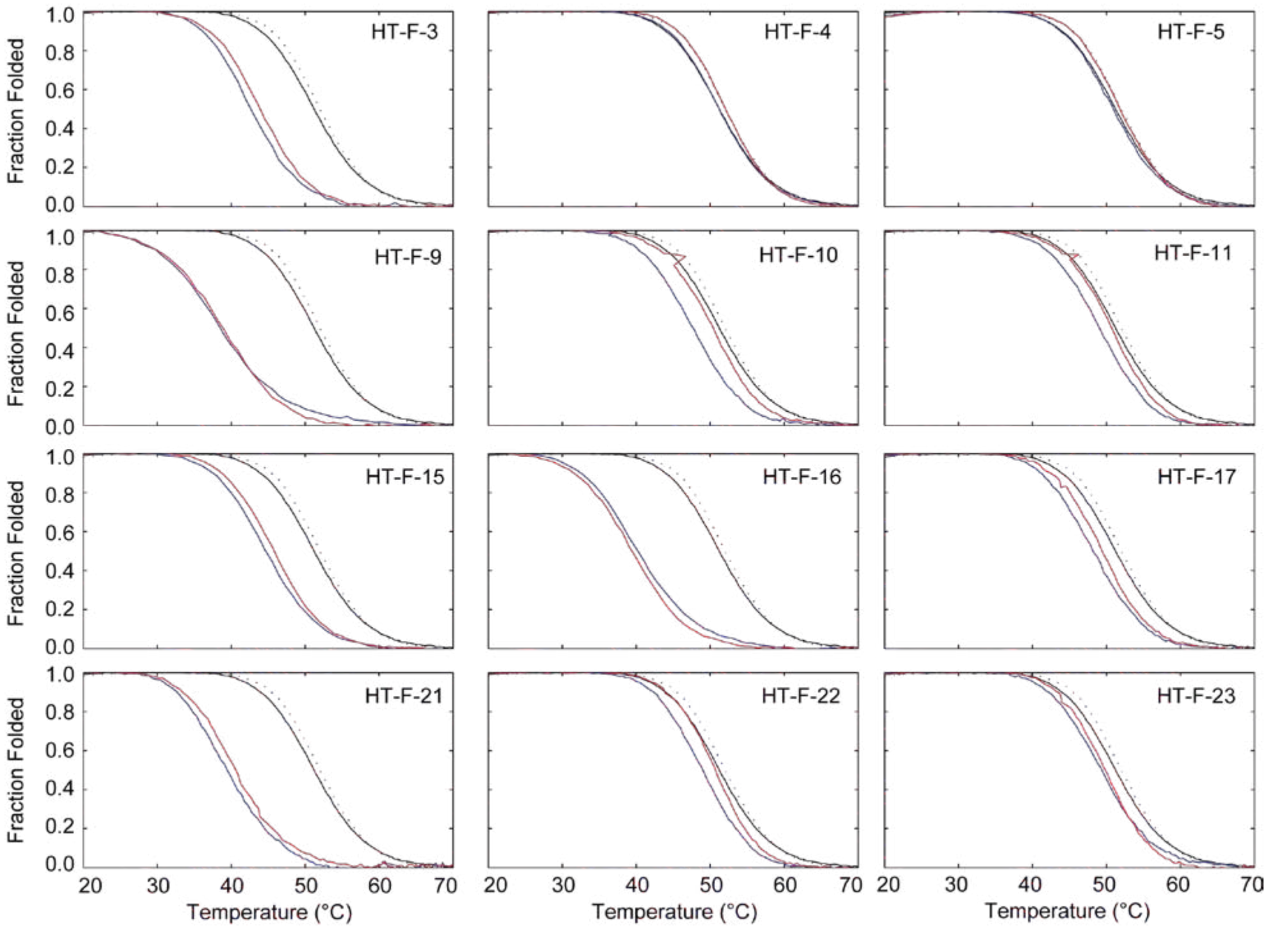

Figure S18: Fraction folded UV spectra of thermal denaturing experiments of ^{F}G-modified HT-series sequences. Heating (red) and cooling (blue) curves are shown. The native sequence (back line) is shown for reference

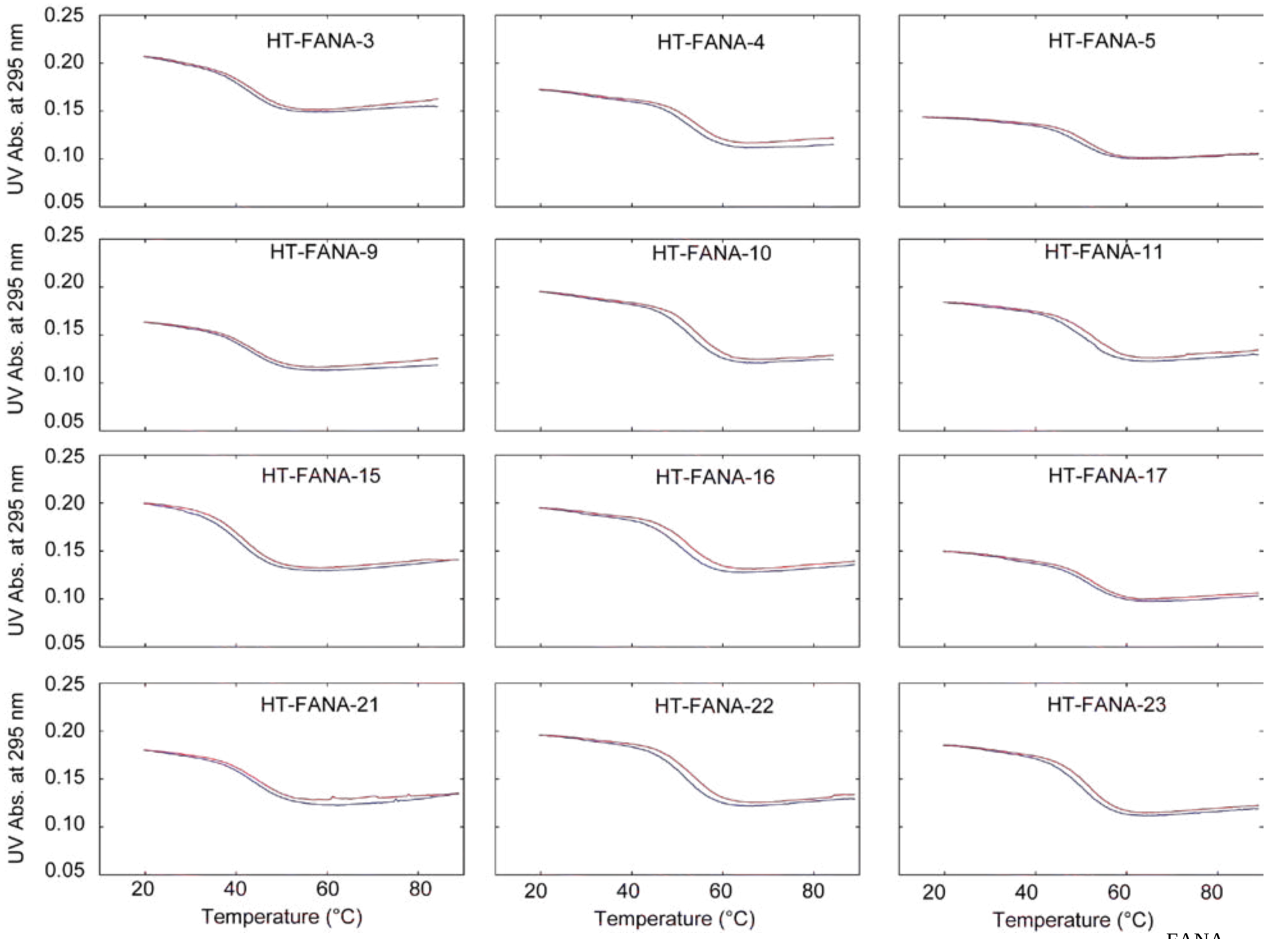

Figure S19: UV absorbance spectra at 295 nm of thermal denaturing experiments of ^{FANA}G-modified (solid line) HT-series sequences. Both heating (red) and cooling (blue) curves are shown.

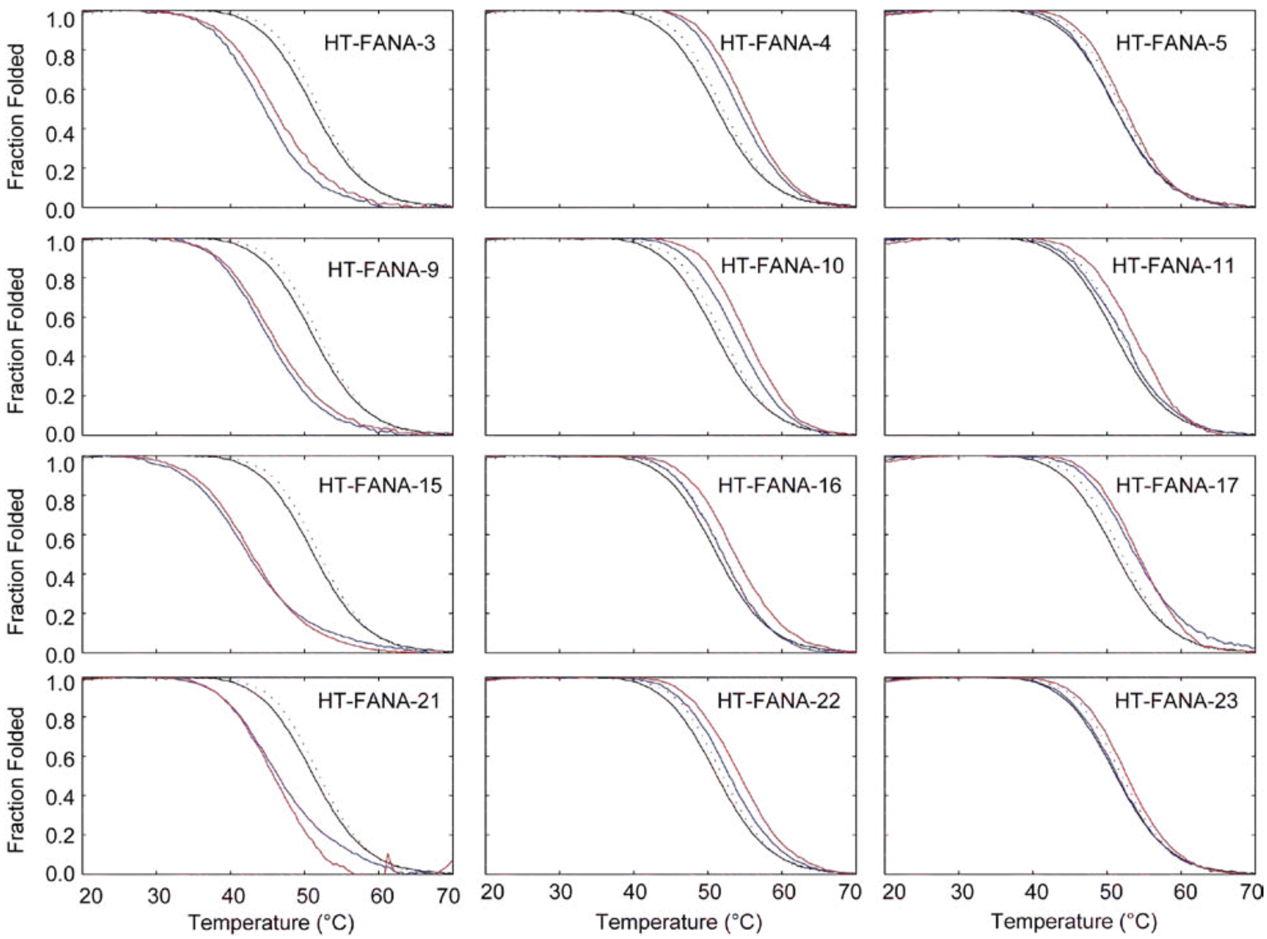

Figure S20: Fraction folded UV spectra of thermal denaturing experiments of ^{FANA}G-modified HT-series sequences. Heating (red) and cooling (blue) curves are shown. The native sequence (back line) is shown for reference

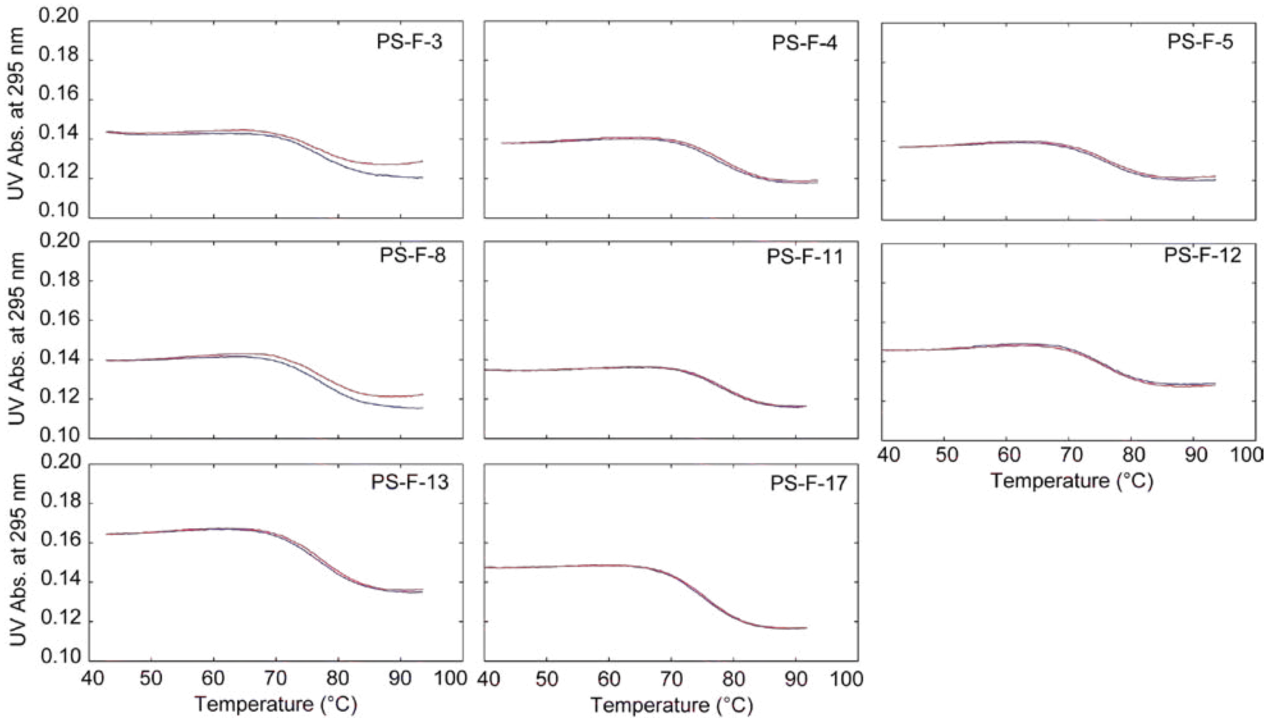

Figure S21: UV absorbance spectra at 295 nm of thermal denaturing experiments of ^{F}G-modified PS-series sequences. Both heating (red) and cooling (blue) curves are shown.

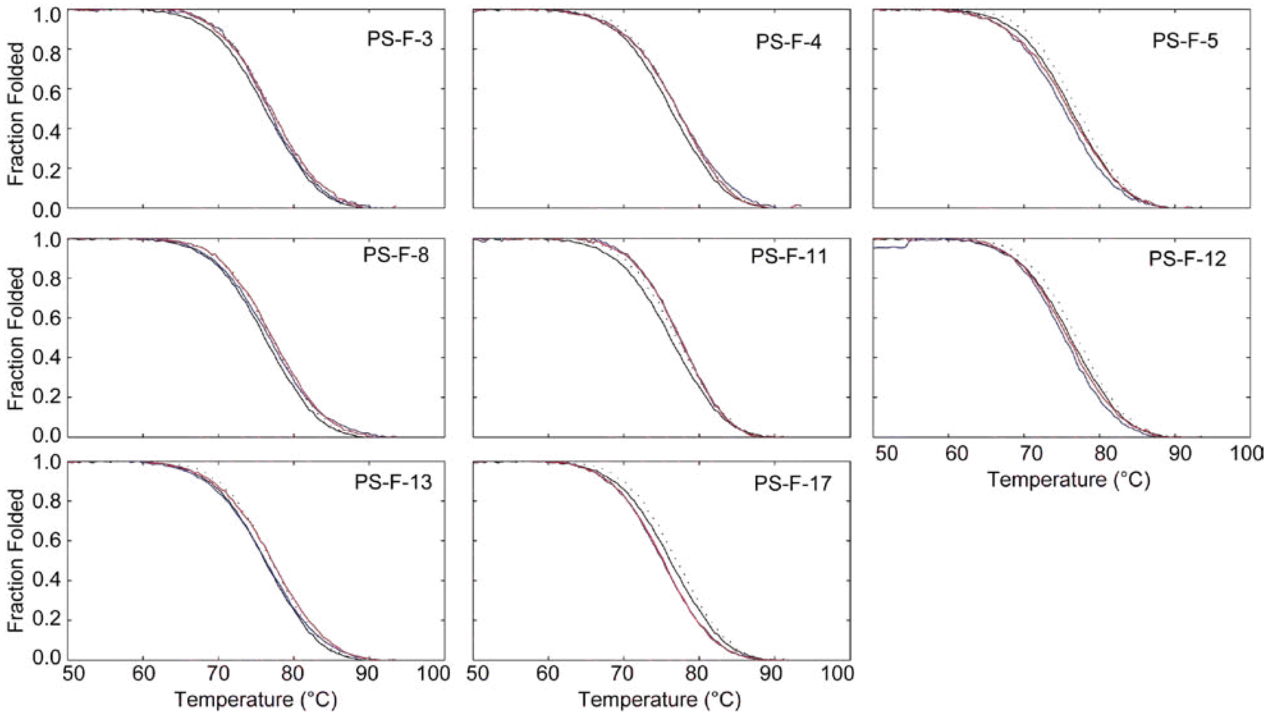

Figure S22: Fraction folded UV spectra of thermal denaturing experiments of ^{F}G-modified PS-series sequences. Heating (red) and cooling (blue) curves are shown. The native sequence (back line) is shown for reference

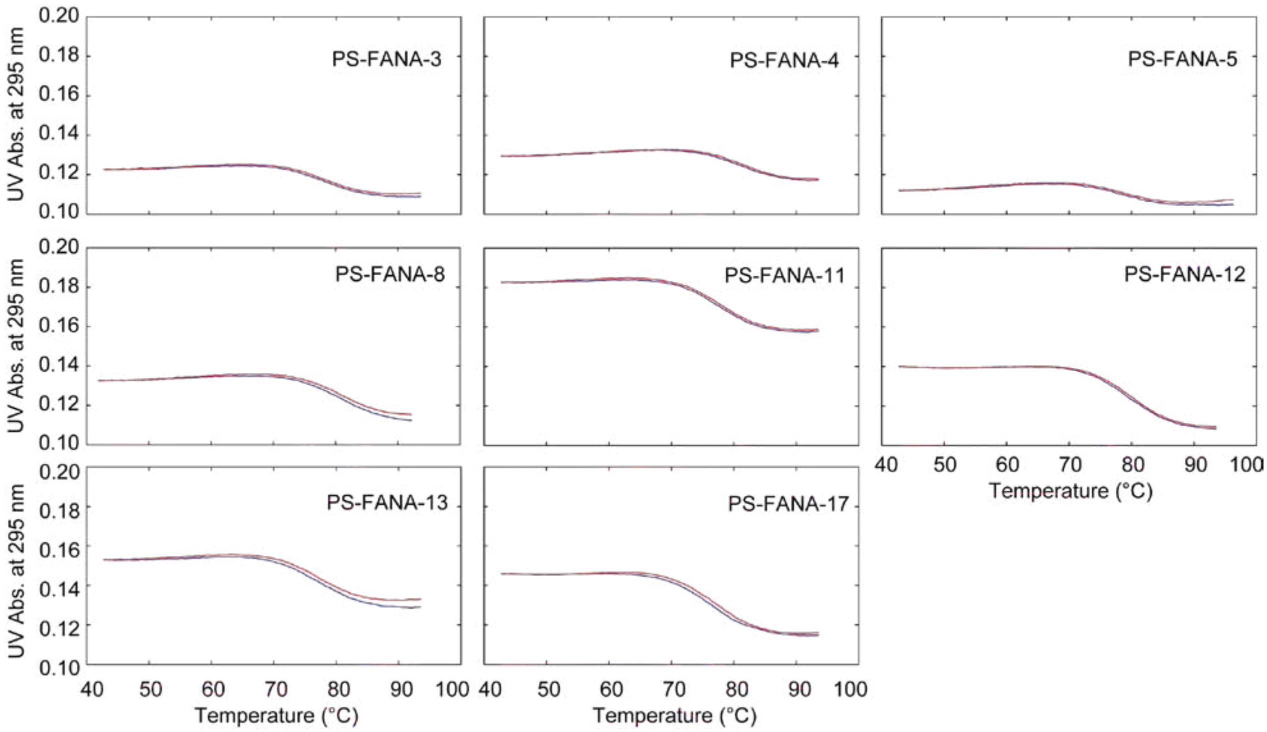

Figure S23: UV absorbance spectra at 295 nm of thermal denaturing experiments of ^{FANA}G-modified PS-series sequences. Both heating (red) and cooling (blue) curves are shown.

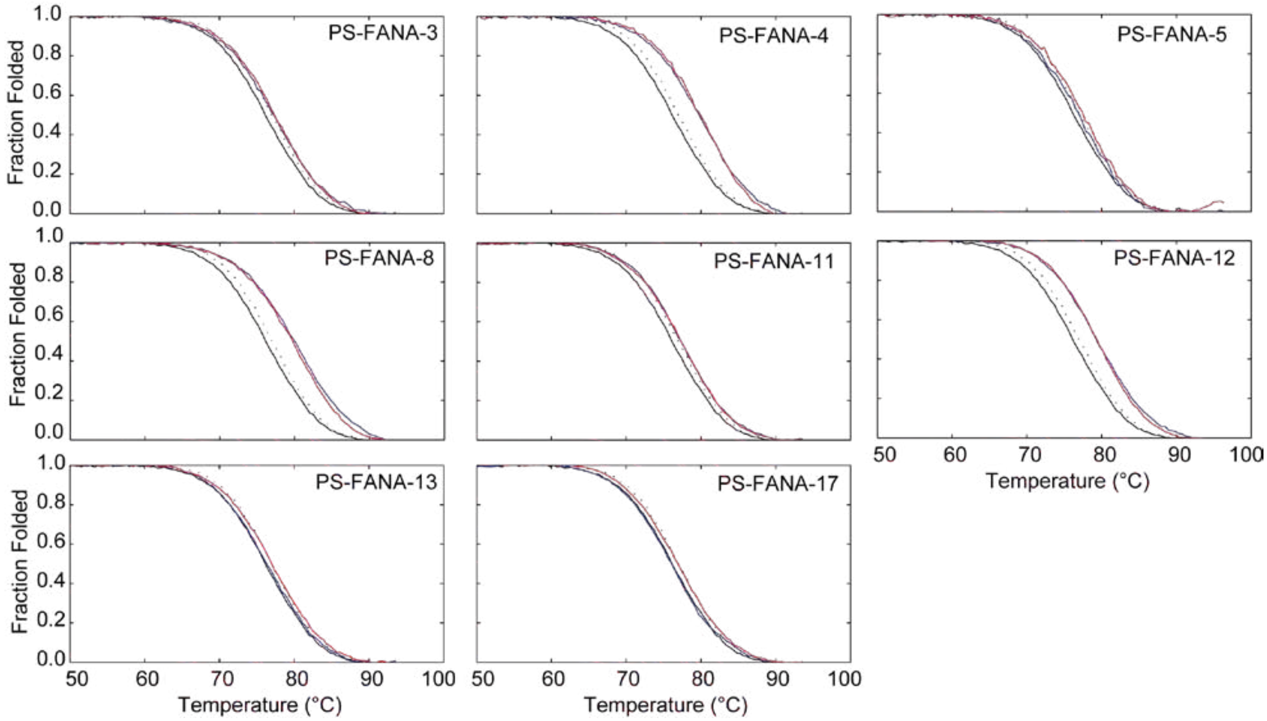

Figure S24: Fraction folded UV spectra of thermal denaturing experiments of ^{FANA}G-modified PS-series sequences. Heating (red) and cooling (blue) curves are shown. The native sequence (back line) is shown for reference
